# Cumulative and variable depression symptom exposure and incident dementia: Panel data analysis of four longitudinal cohort studies

**DOI:** 10.1002/alz.70950

**Published:** 2025-11-28

**Authors:** Zhiyong Hou, Yu Ming, Keqiang Lu, Yiming Ma, Ran Jia, Guichuan Lai, Yuqi Bai, Weijun Huang

**Affiliations:** ^1^ Institute of Basic Research in Clinical Medicine China Academy of Chinese Medical Sciences Beijing PR China; ^2^ Shuguang Clinical Medical College Shanghai University of Traditional Chinese Medicine Shanghai PR China; ^3^ Department of Health Statistics, School of Public Health Chongqing Medical University Chongqing China; ^4^ School of Environment, Education and Development The University of Manchester Manchester UK; ^5^ Department of Otorhinolaryngology Head and Neck Surgery Shanghai Sixth People's Hospital Affiliated to Shanghai Jiao Tong University School of Medicine Shanghai China; ^6^ Otolaryngology Institute of Shanghai Jiao Tong University Shanghai China

**Keywords:** CHARLS, dementia, depression symptoms, ELSA, HRS, longitudinal cohort, SHARE

## Abstract

**INTRODUCTION:**

Late‐life depression symptoms are implicated in dementia. We examined how cumulative burden, duration, trajectory, and variability of depression symptoms are associated with incident dementia.

**METHODS:**

A prospective cohort analysis of 23,305 dementia‐free adults from four population studies (English Longitudinal Study of Ageing [ELSA], Health and Retirement Study [HRS], Survey of Health, Ageing and Retirement in Europe [SHARE], China Health and Retirement Longitudinal Study [CHARLS]) with repeated Centre for Epidemiologic Studies of Depression scale (CES‐D)/Euro‐Depression scale (EURO‐D) assessments across three waves and a pooled median follow‐up of ≈10.8 years. Exposures included CumSD/cumulative average depression symptom score (CumADS), high‐symptom exposure duration, visit‐to‐visit variability (Standard deviation [SD], coefficient of variation [CV], variation independent of the mean [VIM]), and time‐course patterns. Associations were analyzed using multivariable‐adjusted Cox regression.

**RESULTS:**

Each 1‐unit increase in cumulative score was associated with a 3%–8% higher dementia hazard across cohorts. Highest versus lowest cumulative quartiles showed markedly elevated risk. Sustained high exposure for 4 years conferred ≈2.7–3.9× greater risk. Higher variability and worsening trajectories were also linked to higher incidence. Associations were robust across subgroups.

**DISCUSSION:**

Persistent and unstable depression symptoms independently predict higher dementia risk, supporting longitudinal mood monitoring and sustained management.

**Highlights:**

- Multi‐cohort study of 23,305 adults (ELSA, HRS, SHARE, CHARLS).
- Cumulative depression burden shows a dose–response with dementia risk.
- Highest versus lowest quartile: dementia hazard up to 18× (HRS).
- Sustained high symptoms (4 years) linked to ≈2.7–3.9× greater risk.
- Visit‐to‐visit variability independently associates with higher dementia risk.

## 1 BACKGROUND

Dementia is a progressive neurodegenerative syndrome characterized by insidious cognitive decline and impaired activities of daily living. It contributes substantially to disability and mortality among older adults, adversely affecting patients’ and families’ quality of life and imposing considerable health care and social care costs.^1^, ^2^, ^3^ Depression, particularly late‐onset depression, has been identified as a significant, modifiable risk factor for dementia.^4^ The Lancet Commission on Dementia recognized a potential bidirectional relationship between depression and dementia, suggesting that depression may serve both as a prodromal manifestation of underlying neurodegeneration and as an independent risk factor for incident dementia.^5^

In the Rotterdam Study, researchers observed that participants with clinically significant depression symptoms at baseline experienced a substantially higher incidence of Alzheimer's disease and other dementias over 5–10 years of follow‐up.^6^ Further support comes from the Multi‐institutional Research in Alzheimer's Genetic Epidemiology (MIRAGE) Study, which found that both early‐ and late‐onset depressive episodes were associated with faster progression to clinical dementia, suggesting that mood disturbances may unmask latent neuropathology or contribute directly to neuronal injury.^7^ Considerable evidence indicates that depression or elevated depression symptoms are associated with accelerated cognitive decline and an increased risk of dementia.^8^, ^9^, ^10^ Research has shown that chronic inflammation, vascular changes, hypothalamic–pituitary–adrenal (HPA) axis dysregulation, and alterations in neurotrophic factors and neurotransmitter systems contribute to the complex mechanisms linking depression and dementia.^11^, ^12^

In recent years, most evidence on the effects of depression on dementia has relied on single time‐point measurements, which may fail to capture sustained physiological vulnerability to dementia. The course of depression is well known to be variable^13^; therefore, a single‐point measurement is insufficient to capture individual fluctuations in depression symptoms over time. Capturing both the intensity and duration of depressive episodes and incorporating cumulative exposure over time may enhance understanding of the relationship between depression and the risk of incident dementia.

Therefore, based on four large population‐based cohorts, the English Longitudinal Study of Ageing (ELSA), the Health and Retirement Study (HRS), the Survey of Health, Ageing and Retirement in Europe (SHARE), and the China Health and Retirement Longitudinal Study (CHARLS), we aimed to quantify the associations between (1) high variability in depression symptoms, (2) long‐term cumulative depression symptoms, (3) exposure duration of high depression symptoms, and (4) the cumulative burden of depression symptoms with the risk of incident dementia. We further assessed whether these associations were influenced by the temporal pattern of depression symptom accumulation.

## 2 METHODS

### 2.1 Data source and study population

Data were collected from four international aging cohorts: the ELSA, the HRS, the SHARE, and the CHARLS. Specifically, we analyzed data from 2010 to 2020, covering Waves 6–8 (2012–2016) for ELSA, Waves 10–12 (2010–2014) for HRS, Waves 6–8 (2016–2020) for SHARE, and Waves 1–3 (2011–2015) for CHARLS.

**FIGURE 1 alz70950-fig-0001:**
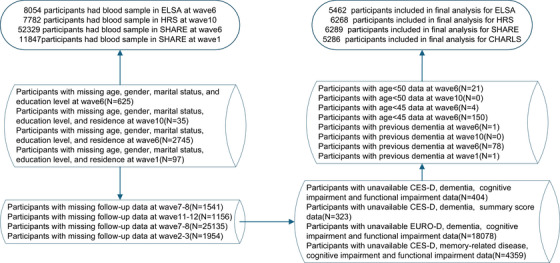
Flow chart of the study population.

The study comprised participants with baseline blood samples from four population‐based cohorts: ELSA (Wave 6, *N* = 8054), HRS (Wave 10, *N* = 7782), SHARE (Wave 6, *N* = 52,329), and CHARLS (Wave 1, *N* = 11,847). In each cohort, we sequentially excluded participants who had: (1) missing sociodemographic information; (2) age below the cohort‐specific threshold (<50 years for ELSA and HRS; <45 years for SHARE and CHARLS); (3) prevalent dementia at baseline; (4) loss to follow‐up over the subsequent two waves; or (5) incomplete Centre for Epidemiologic Studies of Depression scale (CES‐D) and Euro‐Depression scale (EURO‐D), cognitive, or functional assessments. This process yielded final analytic samples of 5462 participants in ELSA, 6268 in HRS, 6289 in SHARE, and 5286 in CHARLS. The study procedure is illustrated in Figure 1. All cohorts received ethical approval from their respective institutional review boards and obtained written informed consent from participants prior to enrollment.

### 2.2 Assessment of depression symptoms and visit‑to‑visit variability

All depression symptom scales were self‐administered. In ELSA and HRS, depression symptoms were assessed with the CES‐D‐8, CHARLS used the CES‐D‐10, and SHARE utilized the EURO‐D scale.^14^, ^15^, ^16^ Raw scores spanned 0–8 for both CES‐D‐8 versions, 0–30 for CES‐D‐10, and 0–12 for EURO‐D. We defined depression symptoms as scores meeting or exceeding specific thresholds (≥3 for HRS and ELSA, ≥10 for CHARLS, and ≥4 for SHARE). In addition, we quantified visit‐to‐visit variability (VVV) of depression symptoms across study visits by calculating the standard deviation (SD), coefficient of variation (CV), and variation independent of the mean (VIM)^17^ to assess symptom stability and evaluate associations between VVV and incident dementia risk. We calculated VIM as 100 × SD / mean^β, where *β* is the regression coefficient from a linear regression of ln(SD) on ln(mean), and computed CV as the ratio of SD to the mean CES‐D/EURO‐D score across visits.

**RESEARCH IN CONTEXT**

1. **Systematic review**: We searched PubMed and Web of Science to identify studies examining the association of depression or elevated depression symptoms with the risk of cognitive decline and dementia. Relatively few population‐based investigations have quantified how long‐term depression exposure—including cumulative burden, exposure duration, temporal patterning, or visit‐to‐visit variability—relates to subsequent dementia risk, and harmonized, cross‐cohort evidence spanning Western and non‐Western populations remains limited.
2. **Interpretation**: Our findings suggest that greater cumulative exposure to depression symptoms, longer periods of high symptom burden, worsening trajectories, and higher within‐person variability in symptoms are each independently associated with incident dementia in a dose‐dependent manner. These associations were consistent across Western and non‐Western cohorts and remained robust after adjustment for sociodemographic, lifestyle, and cardiometabolic covariates, suggesting that both the magnitude and the temporal patterning of depression symptoms matter for long‐term cognitive health.
3. **Future directions**: (1) Clarify causality using randomized trials, quasi‐experimental designs, and mediation analyses that link depression accumulation to biological pathways (e.g., inflammation, hypothalamic‐pituitary‐adrenal [HPA] axis dysfunction, and neurodegeneration). (2) Evaluate whether sustained depression treatment reduces dementia incidence in pragmatic and effectiveness trials. (3) Examine genetic and social moderators, such as apolipoprotein E (*APOE*) ε4 status, markers of cognitive reserve, and socioeconomic factors, to identify subgroups most likely to benefit from prevention. (4) Translate findings into practice by testing scalable longitudinal mood‐monitoring systems and targeted prevention strategies across diverse health care settings and care pathways.

### 2.3 Cumulative depression symptoms and its time course

Cumulative depression symptoms (CumDS) were defined as the summed average CES‐D/EURO‐D at each pair of successive[Fig alz70950-fig-0001] visits, multiplying that average by the interval in years between those visits, and summing across all intervals. We derived the cumulative average depression symptom score (CumADS) by dividing CumDS by the total follow‐up duration. High depression symptom exposure duration was defined as the times of visits with a high CES‐D/EURO‐D score (over the cutoff calculated via an outcome‐oriented method to maximized log‐rank statistics) among the two visits, quantified as 0, 2, and 4 years. To characterize symptom‐accumulation trajectories, we fitted a linear regression of CES‐D/EURO‐D scores against time (years since the first visit) across all three waves; the resulting slope indicated whether depression symptoms increased or decreased over time. Patterns of change in depression symptoms across the three time points were classified as decrease–decrease, decrease–increase, increase–decrease, or increase–increase.

### 2.4 Assessment of dementia

These cohorts conducted cognitive tests, including memory, temporal orientation, and executive function tests. Memory assessments were uniform across the four cohorts, whereas temporal orientation and executive function measures differed. Memory was assessed via immediate and delayed recall of 10 unrelated words in ELSA, HRS, SHARE, and CHARLS. The total memory score was 20 points, which was the sum of the immediate and delayed word recall scores, with 1 point for each word. For temporal orientation in ELSA, SHARE, and CHARLS, participants were asked to recall the day of the week, month, and year, yielding an orientation score (0–4) based on 1 point per correctly recalled item. For executive function, CHARLS administered the serial‐sevens subtraction test (0–5), whereas ELSA and SHARE used animal‐naming fluency. HRS assessed executive function via backward counting and the serial‐sevens subtraction test, with a combined score range of 0–7.

Dementia was defined either by self‐reported physician diagnosis or via validated algorithmic criteria incorporating cognitive and functional assessments.^18^, ^19^, ^20^ The occurrence of dementia was assessed by the following questions: “Has a doctor told you that you have dementia, senility or any other serious memory impairment?” for ELSA and HRS; Has a doctor told you that you have Alzheimer's disease, dementia, or other serious memory impairment?‘’ for SHARE. In HRS, dementia was defined by a cognition summary score (0–27), with scores ≤6 indicating dementia; this summary score encompassed memory and executive function domains. In ELSA, SHARE, and CHARLS, dementia was defined as the concurrence of cognitive impairment and functional dependency.^21^, ^22^ Cognitive impairment was defined as a score ≥1.5 SD below the education‐stratified population mean in any cognitive domain. Functional impairment was defined as self‐reported difficulty with at least one activity of daily living, including bathing, eating, dressing, transferring, or ambulating across a room. Because ascertainment combined algorithmic thresholds and self/proxy reports rather than clinical adjudication, we refer to outcomes as “probable dementia” (“dementia” hereafter denotes probable dementia).

### 2.5 Covariates

The following covariates were collected in this study. (I) Demographic data: age, gender (“Male,” “Female”), marital status (“Married,” “Others”), education level (“College or above,” “High school,” “Below high school”), residence (“Rural,” “Urban”); (II) lifestyle information: smoking status (“Current Smoker,” “Former Smoker,” “Never Smoked”); drinking status; (III) physical examinations: systolic blood pressure (SBP), diastolic blood pressure (DBP), body mass index (BMI); and (IV) previous medical history: depression, hypertension, diabetes, heart disease, stroke; (V) laboratory examinations: C‐reactive protein (CRP), glycosylated hemoglobin (HbA1c), total cholesterol (TC), high‐density lipoprotein cholesterol (HDL‐C). All comorbidities were defined based on a self‐reported history provided by the participants.

### 2.6 Statistical analysis

We employed descriptive statistics to summarize the baseline characteristics of the study participants. Continuous variables with normal distribution were presented as mean ± SD, and continuous variables with non‑normal distribution were presented as median (interquartile range [IQR]). Categorical variables were presented as counts (*N*) and percentages (%). The univariate and multivariate Cox proportional hazards regression models were performed to investigate the associations of the depression symptoms with incident dementia, with representation by hazard ratios (HRs) and 95% confidence intervals (95% CIs) and adjustment for the relevant variables in the model. Four models were estimated: Model 1 did not adjust variables; Model 2 adjusted for age, gender, marital status, education level, and residence; Model 3 adjusted for variables in Model 2 and smoking status, drinking status, CRP, HbA1c, TC, and HDL‐C; and Model 4 adjusted for variables in Model 3 and hypertension, diabetes, heart disease, and stroke. CumDS and CumADS were included in each model both as continuous variables and as quartile‐based categorical variables. Similarly, cumulative burden, exposure duration, slope, and time‐course patterns were incorporated. The *p*‐values for the trend were calculated using the median CumDS, CumADS, and exposure duration in each quartile.

We established Restricted Cubic Spline (RCS) models based on four adjusted Cox regression models, following the Bayesian Information Criterion for model selection to explore the potential nonlinear association of the CumADS with dementia incidence. For each participant, we computed follow‐up time using ages recorded at each wave. For cohorts with three waves, CumDS and CumADS were computed as the time‐weighted sum of paired‐visit averages; follow‐up time for Cox models was implemented as age at event/censor minus age at baseline (delayed‐entry used baseline age). Multiple interpolation is implemented by the Template method (R package “VIM”) ^23^ and the multiple interpolation method (R package “mice”).^24^ In addition, subgroup analyses were performed to investigate whether the relationships between the CumADS and dementia varied according to the status of the covariates (age, gender, marital status, education level, smoking status, drinking status, hypertension, diabetes, heart disease, stroke).

To aid interpretation of extreme relative hazards observed in certain cohort‐specific quartile comparisons, we generated Kaplan–Meier (KM) survival curves stratified by cohort‐specific cumulative depressive‐symptom quartiles. KM curves were estimated using the survfit function (R package “survival”) and compared across quartiles using the log‐rank test. These descriptive KM analyses complement Cox‐model HR estimates by presenting absolute event probabilities and facilitating interpretation when extreme relative estimates may reflect sparse events in the exposure distribution.

To reduce the potential for reverse causation from prodromal dementia, we conducted complementary lagged analyses. Specifically, we excluded participants newly classified with dementia at the second study wave (i.e., within the first inter‐wave interval, ≈2 years in cohorts), re‐estimated the primary Cox proportional‐hazards models in the remaining at‐risk sample, and compared results from the lagged analyses with the primary estimates to assess the influence of early incident cases and the robustness of our findings to potential prodromal effects. Finally, within each cohort, we conducted complete‐case logistic regression sensitivity analyses to assess the robustness of our findings. To address differences in depression instruments and score ranges, we performed sensitivity analyses using z‐standardized depressive‐symptom scores within each cohort (each score was mean‐centered and divided by the cohort standard deviation; *z* = (*x* − mean)/SD). All statistical analyses were performed in R (version 4.4.2). Two‐sided *p*‐values < 0.05 were considered statistically significant.

## 3 RESULTS

### 3.1 Baseline characteristics of participants in all cohorts

Baseline characteristics of the 23,305 participants across four cohorts are summarized in Table 1: 5462 in ELSA, 6268 in HRS, 6289 in SHARE, and 5286 in CHARLS. Median baseline age was 65.0 years (IQR 60.0–72.0) in ELSA, 63.0 years (IQR 56.0–73.0) in HRS, 68.0 years (IQR 63.0–75.0) in SHARE, and 57.0 years (IQR 50.0–63.0) in CHARLS. Educational attainment differed markedly across cohorts: college education or higher was reported by 47.0% in ELSA, 41.0% in HRS, 30.0% in SHARE, and 4.3% in CHARLS. Health behaviors and residential patterns varied by cohort. Current smoking prevalence was 11.0% in ELSA, 15.0% in HRS, 49.0% in SHARE, and 33.0% in CHARLS; alcohol use prevalence ranged from 22.0% in SHARE to 89.0% in ELSA. Median BMI was highest in HRS (29.0 kg/m^2^, IQR 26.0–34.0) and lowest in CHARLS (23.5 kg/m^2^, IQR 21.2–26.1). Cardiovascular and metabolic comorbidity profiles also differed: hypertension prevalence ranged from 27.0% in CHARLS to 56.0% in HRS; diabetes prevalence was 9.7% in ELSA, 21.0% in HRS, 15.0% in SHARE, and 6.7% in CHARLS. Heart disease prevalence ranged from 17.0% to 19.0% across cohorts, whereas stroke history ranged from 2.1% to 5.8%. The prevalence of elevated depression, defined by cohort‐specific CES‐D or EURO‐D thresholds, was 18.0% in ELSA, 21.0% in HRS, 23.0% in SHARE, and 32.0% in CHARLS. Median CRP ranged from 1.03 mg/L (IQR 0.55–2.10) in CHARLS to 2.04 mg/L (IQR 1.47–3.09) in SHARE. Median TC was 5.20 mmol/L (IQR 3.70–6.10) in ELSA and 224 mg/dL (IQR 208–240) in SHARE; median HDL‐C ranged from 49 mg/dL (IQR 40–59) in CHARLS to 69 mg/dL (IQR 63–75) in SHARE. Median HbA1c was 38 mmol/mol (IQR 32–41) in ELSA and 5.96% (IQR 5.71–6.25) in SHARE.

**TABLE 1:** Baseline characteristics of participants in all cohorts.

	ELSA	HRS	SHARE	CHARLS
Characteristic	*N* = 5462^a^	*N* = 6268^a^	*N* = 6289^a^	*N* = 5286^a^
Gender				
Male	2482 (45%)	2572 (41%)	2677 (43%)	2804 (53%)
Female	2980 (55%)	3696 (59%)	3612 (57%)	2482 (47%)
Age, years	65 (60, 72)	63 (56, 73)	68 (63, 75)	57 (50, 63)
Marital status				
Others	1771 (32%)	2514 (40%)	1,846 (29%)	643 (12%)
Married	3691 (68%)	3754 (60%)	4443 (71%)	4643 (88%)
Education level				
Below high school	1659 (30%)	301 (4.8%)	1480 (24%)	4525 (86%)
College or above	2550 (47%)	2558 (41%)	1865 (30%)	229 (4.3%)
High school	1253 (23%)	3409 (54%)	2944 (47%)	532 (10%)
Smoking status				
Current smoker (Yes)	595 (11%)	912 (15%)	3030 (49%)	1753 (33%)
Former smoker	2774 (51%)	2478 (40%)	–	535 (10%)
Never smoked (No)	2092 (38%)	2846 (46%)	3136 (51%)	2997 (57%)
Drinking status	4503 (89%)	3709 (59%)	1359 (22%)	1832 (35%)
Residence				
Rural	–	1594 (25%)	1822 (29%)	1212 (23%)
Urban	–	4674 (75%)	4467 (71%)	4074 (77%)
BMI	27.6 (24.9, 31.0)	29 (26, 34)	26.2 (23.8, 29.1)	23.5 (21.2, 26.1)
SBP	132 (121, 145)	129 (116, 142)	–	126 (114, 140)
DBP	75 (68, 82)	80 (72, 88)	‐	75 (67, 83)
Hypertension	2199 (40%)	3493 (56%)	3251 (52%)	1396 (27%)
Diabetes	529 (9.7%)	1306 (21%)	912 (15%)	350 (6.7%)
Heart disease	943 (17%)	1216 (19%)	1106 (18%)	695 (13%)
Stroke	179 (3.3%)	364 (5.8%)	342 (5.4%)	111 (2.1%)
Depression	982 (18%)	1285 (21%)	1434 (23%)	1704 (32%)
CRP	1.10 (0.30, 2.50)	1.8 (0.8, 3.8)	2.04 (1.47, 3.09)	1.03 (0.55, 2.10)
TC	5.20 (3.70, 6.10)	195 (173, 218)	224 (208, 240)	191 (167, 215)
HDL‐C	1.40 (1.00, 1.80)	55 (46, 64)	69 (63, 75)	49 (40, 59)
HbA1c	38 (32, 41)	5.80 (5.50, 6.30)	5.96 (5.71, 6.25)	5.10 (4.90, 5.40)

Abbreviations: CRP, C‐reactive protein; HbA1c, glycosylated hemoglobin; HDL‐C, high‐density lipoprotein cholesterol; TC, total cholesterol.

^a^ *n* (%); Median (Q1, Q3).

### 3.2 The relationship between cumulative exposure and incident dementia

In fully adjusted Cox models (Model 4; Table 2), higher cumulative depression symptoms exposure was robustly and dose‐dependently associated with higher dementia incidence across all four cohorts (all *p*‐trend < 0.001). Specifically, each 1‐point increment in cumulative score conferred a 3% to 8% (ELSA 1.06, 95% CI 1.04–1.08; HRS 1.08, 1.07–1.10; SHARE 1.08, 1.06–1.09; CHARLS 1.03, 1.03–1.04) increase in risk. Compared with the lowest quartile, participants in the highest quartile exhibited 2.7‐fold (ELSA; HR 2.71, 1.56–4.69) to 18.0‐fold (HRS; HR 18.0, 6.35–51.0) greater risk (all *p*‐trend < 0.001). Those with any positive cumulative burden (≥0) faced 2.4‐ to 5.1‐fold elevations in hazard (ELSA HR 2.36, HRS HR 5.10, SHARE HR 2.80, CHARLS HR 3.55; all *p*’s < 0.001). Sustained high symptom exposure for 4 years versus none yielded 2.7‐ to 3.9‐fold higher risk (ELSA HR 2.69, HRS HR 3.60, SHARE HR 3.89, CHARLS HR 3.82; all *p*‐trend < 0.001). Trajectory analyses showed that worsening patterns, particularly “increase–increase” (HRs up to 3.27 in HRS) and “decrease–increase” (HRs up to 2.94 in HRS; 1.67 in SHARE), were associated with the greatest hazards, whereas the slope of symptom progression reached significance only in CHARLS (HR 1.37; 95% CI 1.04–1.80; *p* = 0.023). These findings persisted across Models 1–3, underscoring that both the magnitude and persistence of depression symptoms independently heighten dementia risk (Tables S1–S4).

**TABLE 2:** Fully adjusted Cox regression models for the association between the cumulative exposure and incident dementia risk.

	ELSA			HRS			SHARE			CHARLS		
Categories	HR	95% CI	*p*	HR	95% CI	*p*	HR	95% CI	*p*	HR	95% CI	*p*
Cumulative CES‐D/EURO‐D	1.06	1.04, 1.08	**<0.001**	1.08	1.07, 1.10	**<0.001**	1.08	1.06, 1.09	**<0.001**	1.03	1.03, 1.04	**<0.001**
Quartile cumulative CES‐D/EURO‐D												
Q1	Reference			Reference			Reference			Reference		
Q2	0.91	0.49, 1.68	0.762	4.07	1.37, 12.1	**0.012**	2.66	1.25, 5.68	**0.011**	1.52	0.85, 2.72	0.161
Q3	1.60	0.90, 2.84	0.107	9.63	3.36, 27.6	**<0.001**	4.72	2.32, 9.61	**<0.001**	2.70	1.58, 4.63	**<0.001**
Q4	2.71	1.56, 4.69	**<0.001**	18.0	6.35, 51.0	**<0.001**	7.35	3.64, 14.8	**<0.001**	7.07	4.27, 11.7	**<0.001**
*p* for trend			**<0.001**			**<0.001**			**<0.001**			**<0.001**
Cumulative average CES‐D/EURO‐D	1.26	1.16, 1.38	**<0.001**	1.39	1.29, 1.49	**<0.001**	1.34	1.25, 1.44	**<0.001**	1.15	1.12, 1.17	**<0.001**
Quartile cumulative average CES‐D/EURO‐D												
Q1	Reference			Reference			Reference			Reference		
Q2	0.91	0.49, 1.68	0.762	4.07	1.37, 12.1	**0.012**	2.66	1.25, 5.68	**0.011**	1.52	0.85, 2.72	0.161
Q3	1.60	0.90, 2.84	0.107	9.63	3.36, 27.6	**<0.001**	4.72	2.32, 9.61	**<0.001**	2.70	1.58, 4.63	**<0.001**
Q4	2.71	1.56, 4.69	**<0.001**	18.0	6.35, 51.0	**<0.001**	7.35	3.64, 14.8	**<0.001**	7.07	4.27, 11.7	**<0.001**
*p* for trend			**<0.001**			**<0.001**			**<0.001**			**<0.001**
Cumulative burden												
<0	Reference			Reference			Reference			Reference		
**≥0**	2.36	1.62, 3.43	**<0.001**	5.10	3.26, 7.98	**<0.001**	2.80	2.02, 3.88	**<0.001**	3.55	2.53, 4.99	**<0.001**
Exposure duration												
0 year	Reference			Reference			Reference			Reference		
2 years	1.47	0.81, 2.66	0.200	2.88	1.77, 4.69	**<0.001**	1.98	1.24, 3.15	**0.004**	1.92	1.28, 2.88	**0.001**
4 years	2.69	1.70, 4.25	**<0.001**	3.60	2.38, 5.42	**<0.001**	3.89	2.61, 5.78	**<0.001**	3.82	2.79, 5.25	**<0.001**
*p* for trend			**<0.001**			**<0.001**			**<0.001**			**<0.001**
Slope												
<0	Reference			Reference			Reference			Reference		
≥0	0.77	0.55, 1.08	0.129	0.72	0.51, 1.04	0.079	1.16	0.87, 1.55	0.299	1.37	1.04, 1.80	**0.023**
Time course patterns												
Decrease‐decrease	Reference			Reference			Reference			Reference		
Decrease‐increase	1.40	0.92, 2.15	0.119	2.94	1.84, 4.69	**<0.001**	1.67	1.12, 2.48	**0.012**	2.22	1.46, 3.36	**<0.001**
Increase‐decrease	1.43	0.93, 2.19	0.099	2.50	1.55, 4.04	**<0.001**	1.36	0.90, 2.05	0.141	2.06	1.34, 3.16	**<0.001**
Increase‐increase	1.37	0.66, 2.85	0.396	3.27	1.70, 6.28	**<0.001**	1.41	0.83, 2.37	0.201	1.99	1.20, 3.28	**0.007**

Abbreviations: CI, confidence interval; CRP, C‐reactive protein; HbA1c, glycosylated hemoglobin; HDL‐C, high‐density lipoprotein cholesterol; HR, hazard ratio; TC, total cholesterol.

Bold values statistically significant *p*‐values (*p* < 0.05).

Adjusted for age, gender, marital status, education level, residence, smoking status, drinking status, CRP, HbA1c, TC, HDL‐C, hypertension, diabetes, heart disease, stroke.

KM curves stratified by cumulative depression symptoms quartile are shown in Figures S1–S4. These KM‐based absolute estimates demonstrate that participants in the highest cumulative‐depression quartile, time‐course patterns, and exposure duration experienced substantially greater absolute dementia incidence over time and help contextualize large relative hazards (for example the HR ≈18 in HRS), which primarily reflect very small numbers of participants/events in extreme exposure strata. Results from competing risk analyses accounting for death were directionally consistent.

**FIGURE 2:**
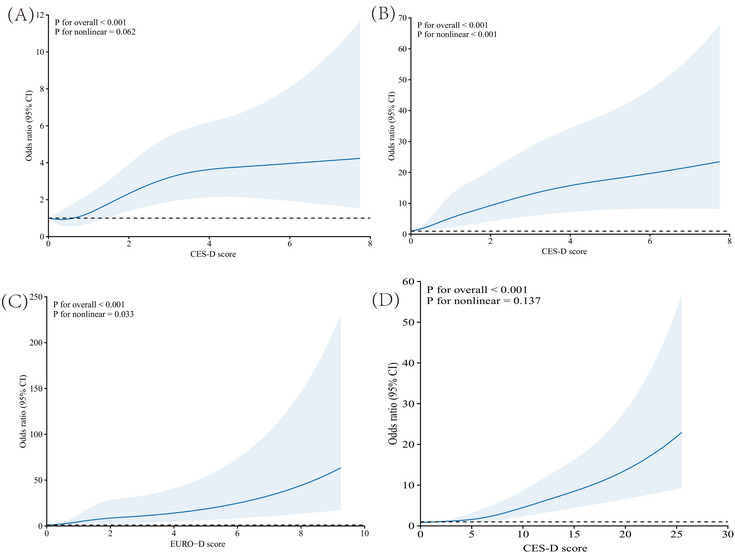
Restricted cubic spline regression. The RCS analysis for full adjustment model in ELSA (A), HRS 1 (B), SHARE (C), and CHARLS (D). All cohorts were with fully adjusted model, including age, gender, marital status, education level, residence, smoking status, drinking status, CRP, HbA1c, TC, HDL‐C, hypertension, diabetes, heart disease, and stroke. CHARLS, China Health and Retirement Longitudinal Study; CRP, C‐reactive protein; ELSA, English Longitudinal Study of Ageing; HbA1c, glycosylated hemoglobin; HDL‐C, high‐density lipoprotein cholesterol; HRS, Health and Retirement Study; RCS, Restricted Cubic Splin; SHARE, Survey of Health, Ageing and Retirement in Europe; TC, total cholesterol.

Across all four cohorts, RCS modeling of CumADS revealed a consistently strong, positive dose–response association with incident dementia in fully adjusted models. In ELSA and CHARLS (Figure 2A, D), the spline curves rose in a near‐linear fashion (*p*‐overall < 0.001; *p*‐non‐linear 0.062 and 0.137, respectively), indicating that each additional point in CumADS steadily increased dementia risk without evidence of a safe threshold. By contrast, HRS exhibited a pronounced J‐shaped pattern (*p*‐overall < 0.001; *p*‐non‐linear < 0.001), with dementia odds remaining flat at low CumADS scores before escalating sharply (Figure 2B). SHARE's EURO‐D spline occupied an intermediate position: risk increased nearly linearly up to CumADS approximately equal to 6 (Figure 2C), then accelerated more steeply at higher scores (*p*‐overall < 0.001; *p*‐nonlinear = 0.033). In all datasets, even modest elevations in CumADS were associated with heightened dementia risk, underscoring the public health imperative to recognize and mitigate depression symptoms across the full exposure spectrum.

### 3.3 Association between VVV of depression symptoms and incident dementia

**FIGURE 3:**
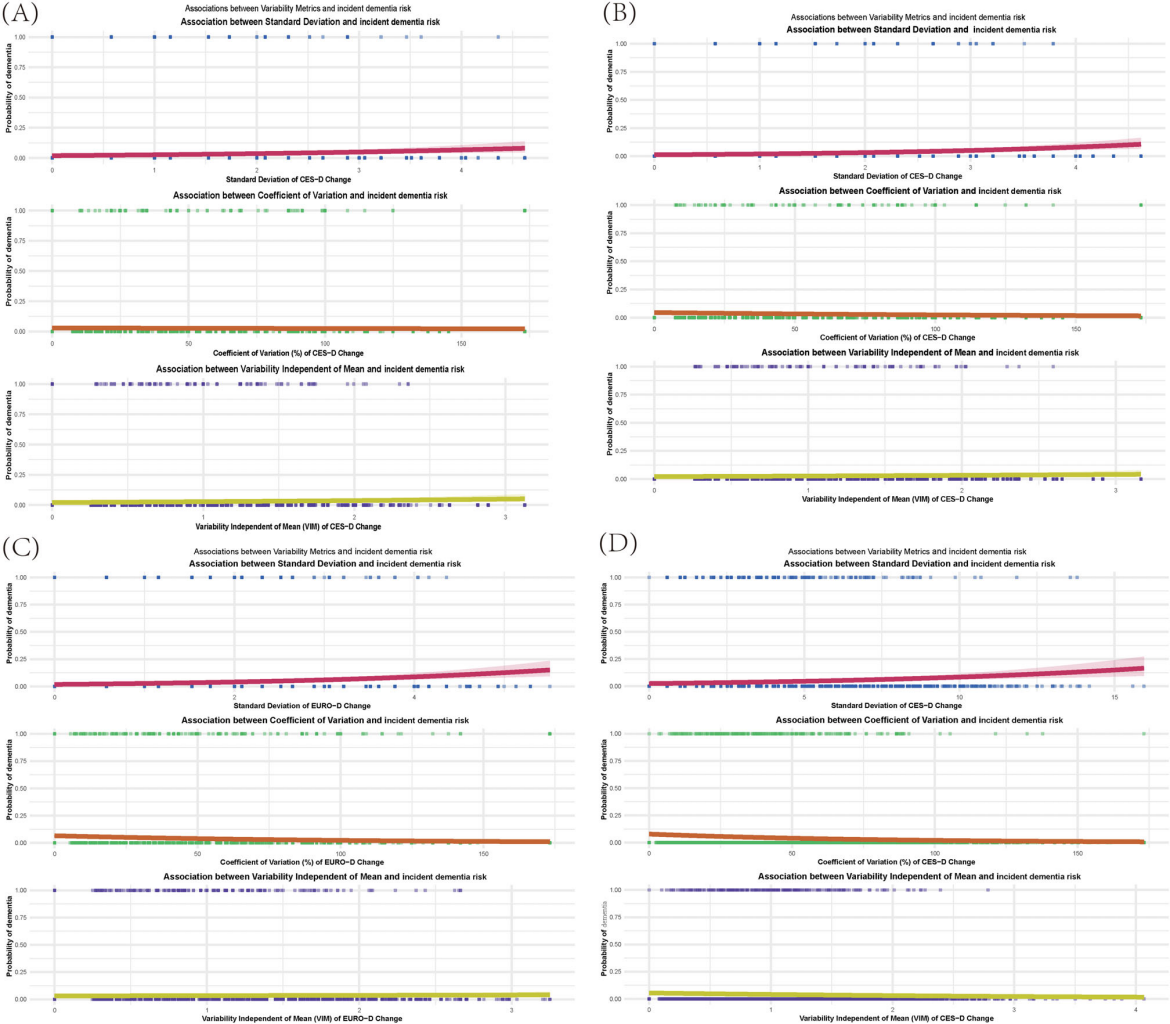
Visit‑to‑visit variability of the depression symptoms and incident dementia risk. The association of SD, CV, and VIM of the depression symptoms with dementia in ELSA (A), HRS 1 (B), SHARE (C), and CHARLS (D). CHARLS, China Health and Retirement Longitudinal Study; CV, coefficient of variation; ELSA, English Longitudinal Study of Ageing; HRS, Health and Retirement Study; SD, Standard deviation; SHARE, Survey of Health, Ageing and Retirement in Europe; VIM, variation independent of the mean.

Across four longitudinal cohorts (ELSA, HRS, SHARE, and CHARLS), within‐participant variability in depression symptoms, quantified by visit‐specific SD, CV, and VIM, varied markedly among individuals. Some participants exhibited tight clustering of scores around their individual mean (low SD and CV), whereas others showed pronounced dispersion (high SD and CV). Scatter‐plot analyses in each cohort revealed positive linear trends: higher SD values corresponded to incremental increases in predicted dementia risk, with analogous patterns for CV. These findings indicate that greater absolute SD and relative CV fluctuations in depression symptoms over time are modestly but consistently linked to elevated dementia risk. In sensitivity analyses employing VIM, the association between VIM and dementia mirrored those observed with SD and CV. Thus, VIM analyses reinforced the SD and CV findings, providing robust evidence that variability in depression symptom trajectories is associated with increased dementia risk (Figure 3A–D). Collectively, these results underscore the importance of assessing visit‐to‐visit variability in depression symptoms as an independent predictor of dementia.

### 3.4 Lagged analyses

To reduce the possibility that prodromal dementia accounted for our findings, we re‐ran the fully adjusted Cox models after excluding participants newly classified as having dementia at the second study wave (Table 3). The principal associations were preserved (Tables S5–S8). Per‐unit associations for cumulative depression symptoms remained significant and of similar magnitude to the primary analysis: ELSA HR 1.06 (95% CI 1.04–1.09), HRS HR 1.09 (95% CI 1.07–1.11), SHARE HR 1.08 (95% CI 1.06–1.09), and CHARLS HR 1.04 (95% CI 1.03–1.05) (all *p*’s < 0.001). Comparisons of extreme burden (Q4 vs Q1) remained elevated in the lagged sample (ELSA HR 3.24 [1.73–6.05]; HRS HR 18.9 [5.79–62.0]; SHARE HR 5.84 [2.86–11.9]; CHARLS HR 7.37 [3.58–15.2]; all *p*’s < 0.001), and cumulative burden (≥0 vs <0) continued to confer substantially higher hazards across cohorts (ELSA 2.44 [1.63–3.64]; HRS 5.07 [3.02–8.53]; SHARE 3.40 [2.36–4.92]; CHARLS 4.16 [2.56–6.76]; all *p*’s < 0.001). Sustained high‐symptom exposure (4 vs 0 years) again showed robust risk increases (ELSA 2.85 [1.71–4.74]; HRS 4.37 [2.70–7.06]; SHARE 4.03 [2.57–6.30]; CHARLS 3.86 [2.49–6.01]; all *p*’s < 0.001). Trajectory analyses produced comparable results: patterns involving increasing symptoms (e.g., HRS increase–increase HR 3.93 [1.85–8.38]; decrease–increase HR 2.85 [1.60–5.07]) remained strongly associated with dementia. Notably, estimates for slope of symptom progression changed modestly: the previously non‐significant slope in HRS became statistically significant in the lagged analysis (slope ≥0: HR 0.63, 95% CI 0.41–0.96, *p* = 0.032), whereas slope effects in other cohorts were unchanged or borderline. Overall, exclusion of early incident cases did not materially attenuate associations, indicating that our principal findings are unlikely to be driven solely by prodromal depression.

**TABLE 3:** Fully adjusted Cox regression models for the association between the cumulative exposure and incident dementia risk with lagged analyses.

	ELSA			HRS			SHARE			CHARLS		
Categories	HR	95% CI	*p*	HR	95% CI	*p*	HR	95% CI	*p*	HR	95% CI	*p*
Cumulative CES‐D/EURO‐D	1.06	1.04, 1.09	**<0.001**	1.09	1.07, 1.11	**<0.001**	1.08	1.06, 1.09	**<0.001**	1.04	1.03, 1.05	**<0.001**
Quartile cumulative CES‐D/EURO‐D												
Q1	Reference			Reference			Reference			Reference		
Q2	1.16	0.59, 2.27	0.670	4.50	1.29, 15.7	**0.018**	2.15	0.99, 4.68	0.053	1.18	0.50, 2.82	0.706
Q3	1.94	1.02, 3.67	**0.042**	7.81	2.29, 26.6	**0.001**	3.86	1.88, 7.95	**<0.001**	3.41	1.61, 7.23	**0.001**
Q4	3.24	1.73, 6.05	**<0.001**	18.9	5.79, 62.0	**<0.001**	5.84	2.86, 11.9	**<0.001**	7.37	3.58, 15.2	**<0.001**
*p* for trend			**<0.001**			**<0.001**			**<0.001**			**<0.001**
Cumulative average CES‐D/EURO‐D	1.28	1.16, 1.42	**<0.001**	1.41	1.30, 1.54	**<0.001**	1.35	1.25, 1.47	**<0.001**	1.16	1.12, 1.20	**<0.001**
Quartile cumulative average CES‐D/EURO‐D												
Q1	Reference			Reference			Reference			Reference		
Q2	1.16	0.59, 2.27	0.670	4.50	1.29, 15.7	**0.018**	2.15	0.99, 4.68	0.053	1.18	0.50, 2.82	0.706
Q3	1.94	1.02, 3.67	**0.042**	7.81	2.29, 26.6	**0.001**	3.86	1.88, 7.95	**<0.001**	3.41	1.61, 7.23	**0.001**
Q4	3.24	1.73, 6.05	**<0.001**	18.9	5.79, 62.0	**<0.001**	5.84	2.86, 11.9	**<0.001**	7.37	3.58, 15.2	**<0.001**
*p* for trend			**<0.001**			**<0.001**			**<0.001**			**<0.001**
Cumulative burden												
<0	Reference			Reference			Reference			Reference		
≥0	2.44	1.63, 3.64	**<0.001**	5.07	3.02, 8.53	**<0.001**	3.40	2.36, 4.92	**<0.001**	4.16	2.56, 6.76	**<0.001**
Exposure duration												
0 year	Reference									Reference		
2 years	1.68	0.91, 3.10	0.097	3.41	1.92, 6.07	**<0.001**	2.31	1.38, 3.87	**0.001**	2.19	1.29, 3.71	**0.004**
4 years	2.85	1.71, 4.74	**<0.001**	4.37	2.70, 7.06	**<0.001**	4.03	2.57, 6.30	**<0.001**	3.86	2.49, 6.01	**<0.001**
*p* for trend			**<0.001**			**<0.001**			**<0.001**			**<0.001**
Slope												
<0	Reference			Reference			Reference			Reference		
≥0	0.73	0.51, 1.05	0.092	0.63	0.41, 0.96	**0.032**	1.28	0.93, 1.77	0.133	1.41	0.97, 2.06	0.071
Time‐course patterns												
Decrease‐decrease	Reference			Reference			Reference			Reference		
Decrease‐increase	1.46	0.93, 2.29	0.102	2.85	1.60, 5.07	**<0.001**	1.73	1.11, 2.70	**0.016**	2.27	1.27, 4.05	**0.006**
Increase‐decrease	1.38	0.87, 2.19	0.175	2.68	1.51, 4.78	**<0.001**	1.42	0.90, 2.25	0.131	2.40	1.34, 4.31	**0.003**
Increase‐increase	1.43	0.65, 3.12	0.373	3.93	1.85, 8.38	**<0.001**	1.36	0.75, 2.47	0.312	1.70	0.82, 3.52	0.152

Abbreviations: CI, confidence interval; CRP, C‐reactive protein; HbA1c, glycosylated hemoglobin; HDL‐C, high‐density lipoprotein cholesterol; HR, hazard ratio; TC, total cholesterol.

Bold values statistically significant *p*‐values (*p* < 0.05).

Adjusted for age, gender, marital status, education level, residence, smoking status, drinking status, CRP, HbA1c, TC, HDL‐C, hypertension, diabetes, heart disease, stroke.

### 3.5 Subgroup analyses and sensitivity analyses

**FIGURE 4:**
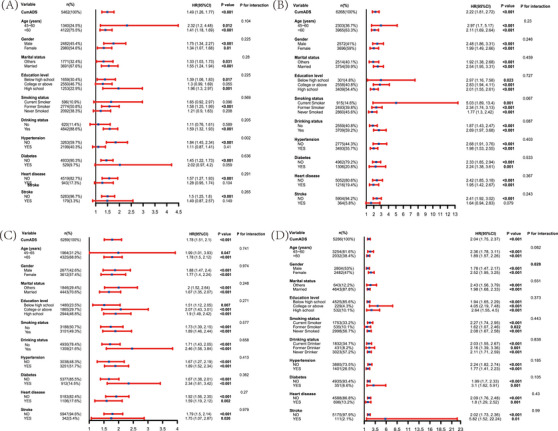
Subgroup analyses. Subgroup analysis for the association of the depressive symptoms and incident dementia risk in ELSA (A), HRS 1 (B), SHARE (C), and CHARLS (D). All cohorts were with fully adjusted model including age, gender, marital status, education level, residence, smoking status, drinking status, CRP, HbA1c, TC, HDL‐C, hypertension, diabetes, heart disease, stroke.

We conducted subgroup analyses within each cohort to evaluate whether the association between CumADS and incident dementia varied according to key demographic and clinical factors (Figure 4A–D). Across strata defined by age (45–60/65 vs ≥60/65 years), gender, marital status, education level, smoking status, alcohol use, hypertension, diabetes, heart disease, and stroke history, HRs for dementia per 1 SD increase in CumADS remained consistently elevated, and none of the tests for interaction were statistically significant (all *p*’s for interaction > 0.05). These findings indicate that the dose–response relationship between cumulative depression symptoms and dementia risk is robust and consistent across diverse population subgroups.

Sensitivity analyses using complete‐case logistic‐regression models corroborated the primary Cox findings (Tables S9–S12). Across the four cohorts, each 1‐unit increase in CumDS and CumADS remained significantly associated with incident dementia: odds ratios (ORs) per 1‐unit increase were 1.07 (95% CI 1.04–1.09) in ELSA, 1.09 (95% CI 1.07–1.12) in HRS, 1.09 (95% CI 1.07–1.11) in SHARE, and 1.04 (95% CI 1.03–1.04) in CHARLS (all *p*’s < 0.001). Quartile analyses demonstrated monotonic dose–response gradients; for example, fully adjusted ORs comparing the highest versus lowest quartile ranged from 2.95 (ELSA) to 20.2 (HRS). Cumulative burden (≥0 vs <0) was associated with ORs ranging from 2.62 (ELSA) to 5.43 (HRS), and exposure duration (≥4 vs 0 years) yielded ORs between 3.02 (ELSA) and 3.65 (HRS). Time‐course patterns characterized by sustained or increasing symptoms were consistently associated with elevated dementia risk across all cohorts. Overall, these sensitivity estimates align with the primary Cox results in both magnitude and direction, confirming the robustness of the observed associations.

To address differences in depression instruments and score ranges across cohorts, we repeated fully adjusted Cox models using cohort‐specific z‐standardized cumulative depressive‐symptom metrics (per 1 SD increase). The direction and significance of associations persisted: per 1 SD increase in CumADS, the pooled HR was 1.18 (95% CI 1.16–1.19). Cohort‐specific contrasts based on *z*‐standardized scores were broadly concordant with the primary analyses (Q4 vs Q1: ELSA HR 2.58, HRS HR 18.1, SHARE HR 7.57, CHARLS HR 6.12; cumulative burden ≥0 vs <0: ELSA HR 2.33, HRS HR 3.18, SHARE HR 2.87, CHARLS HR 3.34). Exposure‐duration effects (4 vs 0 years) likewise remained robust (*z*‐standardized HRs 3.08–3.94 across cohorts). These results indicate that our findings are not driven by differences in depression instruments or scale ranges and that the observed associations are robust to harmonization by standardization (Tables S13–S16).

## 4 DISCUSSION

In this large multinational analysis, we found that long‐term patterns of depression symptoms significantly predict incident dementia. Across four cohorts totaling over 23,000 older adults, higher CumDS/CumADS, longer duration of high‐symptom exposure, and greater visit‐to‐visit symptom variability were each associated with significantly elevated dementia risk. These associations remained robust after adjustment for a wide range of covariates. We observed clear dose–response trends: each incremental increase in CumDS/CumADS raised dementia hazard, and individuals in the highest exposure categories had a several‐fold higher risk compared to those with minimal symptoms. Notably, participants whose depression symptoms remained high or worsened over time (“increase–increase” trajectories) faced the greatest hazards, underscoring that chronicity and persistence of mood disturbance are especially detrimental. These relationships were evident whether exposure was modeled continuously, by quartile, by binary cumulative burden, or by trajectory pattern, and persisted after extensive adjustment for demographics and baseline risk.

In three major capacities, the results we provide add to the present research: First, rather than depending on a single cross‐sectional assessment, we deployed repeated measures to capture the temporal accumulation and magnitude of depression symptoms. All of this allowed us to demonstrated that dementia risk fluctuates by persistence and chronicity instead of merely point prevalence. Second, we indicated the generalization of the cumulative‐exposure effect across Western and Eastern populations by harmonizing analyses across ELSA, HRS, SHARE, and CHARLS. Third, inclusion of variability metrics (SD, CV, VIM) and time‐course patterns provides novel evidence that higher fluctuation and unfavorable trajectories, particularly persistent or worsening symptoms, substantially increase long‐term dementia risk beyond mean symptom levels.

Heterogeneity in the shape and magnitude of associations across cohorts warrants careful interpretation. Restricted cubic spline analyses indicated near‐linear risk increases in ELSA and CHARLS but pronounced nonlinearity (J‐shaped/accelerating patterns) in HRS and SHARE. Several factors may account for these differences: (1) measurement differences (CES‐D‐8, CES‐D‐10, EURO‐D) and score distributions alter the exposure scale and dynamic range; (2) cohort‐specific case ascertainment and follow‐up procedures may influence absolute hazard estimates; and (3) cultural variation in symptom reporting, help‐seeking, or access to diagnosis may accentuate extreme‐score effects in some samples. Extremely large HRs in the highest quartiles (e.g., HRS) likely reflect sparse data at the extreme tail of the exposure distribution and should be interpreted as indicative of markedly elevated risk rather than precise fold changes. Crucially, despite such variation in curve shape and effect size, the directionality and statistical significance of the associations were consistent across cohorts, reinforcing the robustness of the cumulative‐depression signal.

Our results are consistent with prior literature linking depression to cognitive decline and dementia. Although Zhu et al. employed area‐under‐the‐curve metrics for depression symptoms in HRS and ELSA, similarly indicating that higher cumulative depression predicted accelerated cognitive decline and increased dementia incidence.^25^ We extend these findings by incorporating two additional cohorts (SHARE and CHARLS) and by evaluating multiple dimensions of symptom patterns. Whereas prior studies often relied on a single depression assessment, our longitudinal approach reveals that both the magnitude and persistence of depression are significant. The very large HRs for the highest symptom quartile (up to ≈18 in one cohort) suggest that sustained depression may be an even stronger risk factor than transient episodes. It aligns with meta‐analytic evidence that depression roughly doubles dementia risk over the life course,^26^, ^27^ whereas our results imply that the effect can be far larger when symptoms are chronic.

Mechanistically, several biological and behavioral mechanisms could underlie this association. Chronic depression is linked to systemic inflammation, cerebrovascular changes, dysregulation of the HPA axis, reduced neurotrophic support (e.g., Brain‐Derived Neurotrophic Factor (BDNF)), and behavioral pathways that lower cognitive reserve (reduced social engagement, physical inactivity, poorer sleep).^28^, ^29^, ^30^ For instance, prolonged elevation of stress hormones and proinflammatory cytokines in depression may accelerate hippocampal atrophy and amyloid deposition, key features of Alzheimer's pathology.^31^, ^32^ Depression also reduces levels of neurotrophic factors and disrupts neurotransmitter systems (e.g., serotonergic, cholinergic) that support cognition.^33^, ^34^, ^35^ Our findings of a dose–response relationship suggest that these mechanisms may accumulate over time: the more sustained and fluctuating the depression exposure, the greater the neuropathological impact. The observed relationships with VVV suggest that not only the burden but instability of mood, possibly reflecting recurrent relapses, inadequate treatment, or fluctuating stressors, may amplify neurobiological stressors that accelerate neurodegeneration. These putative pathways mirror findings from other cumulative‐exposure fields,^36^, ^37^ where duration and accumulation predict downstream disease more strongly than single measurements.

We acknowledge several important limitations. The observational design precludes definitive causal inference. Although lagged analyses indicate that reverse causation is unlikely to fully account for the observed relationships. Nevertheless, because prodromal neurodegeneration can precede clinical diagnosis by several years, residual reverse causality cannot be completely excluded. Depression symptoms were self‐reported and assessed at only three waves, which may under‐represent intermittent episodes occurring between assessments and could bias effect estimates toward the null (i.e., attenuate observed associations). Dementia ascertainment relied on algorithmic criteria and, where available, self‐ or proxy‐reported physician diagnosis rather than uniform clinical adjudication. Such algorithms can be biased by education, cultural factors, and test availability and do not distinguish neuropathological subtypes. We lacked harmonized data across all cohorts on antidepressant medication use, lifetime psychiatric history, anxiety disorders, and *APOE* genotype; residual confounding by these factors is therefore possible. Finally, cohort differences in depression‐scale versions, follow‐up timing, and cultural patterns of symptom reporting may influence point estimates and spline shapes; nonetheless, the overall pattern of association was consistent across cohorts.

## 5 CONCLUSION

Across four international cohorts, we demonstrate that not only the presence but also the long‐term burden, persistence, and variability of depression symptoms independently predict incident dementia; these associations exhibit a clear dose–response relationship with no apparent safe threshold. Collectively, these results reinforce depression as a potentially modifiable dementia risk factor and support routine, longitudinal monitoring of mood in midlife and late life. Whether interventions that reduce chronic depression burden can lower dementia incidence remains to be tested. Nonetheless, our findings support integrating sustained depression care into population‐level strategies for dementia prevention.

## CONFLICT OF INTEREST STATEMENT

The authors declare no competing interests. Any author disclosures are available in the Supporting Information.

## Supporting information



Supporting Information

Supporting Information

## References

[1] 2023 Alzheimer's disease facts and figures. Alzheimers Dement. 2023;19(4):1598‐1695. doi:10.1002/alz.13016 36918389

[2] Liu Y , Wu Y , Chen Y , et al. Projection for dementia burden in China to 2050: a macro‐simulation study by scenarios of dementia incidence trends. Lancet Reg Health West Pac. 2024;50:101158. doi:10.1016/j.lanwpc.2024.101158 39185089 PMC11342197

[3] Brayne C , Miller B . Dementia and aging populations‐A global priority for contextualized research and health policy. PLoS Med. 2017;14(3):e1002275. doi:10.1371/journal.pmed.1002275 28350794 PMC5370112

[4] Gatchel JR , Rabin JS , Buckley RF , et al. Longitudinal Association of depression symptoms with cognition and cortical amyloid among community‐dwelling older adults. JAMA Netw Open. 2019;2(8):e198964. doi:10.1001/jamanetworkopen.2019.8964 31397865 PMC6692684

[5] Livingston G , Huntley J , Liu KY , et al. Dementia prevention, intervention, and care: 2024 report of the Lancet standing Commission. Lancet. 2024;404(10452):572‐628. doi:10.1016/S0140-6736(24)01296-0 39096926

[6] Mirza SS , Wolters FJ , Swanson SA , et al. 10‐year trajectories of depressive symptoms and risk of dementia: a population‐based study. Lancet Psychiatry. 2016;3(7):628‐635. doi:10.1016/S2215-0366(16)00097-3 27138970

[7] Green RC , Cupples LA , Kurz A , et al. Depression as a risk factor for Alzheimer disease: the MIRAGE Study. Arch Neurol. 2003;60(5):753‐759. doi:10.1001/archneur.60.5.753 12756140

[8] Stephan BCM , Cochrane L , Kafadar AH , et al. Population attributable fractions of modifiable risk factors for dementia: a systematic review and meta‐analysis. Lancet Healthy Longev. 2024;5(6):e406‐e421. doi:10.1016/S2666-7568(24)00061-8 38824956 PMC11139659

[9] Bennett S , Thomas AJ . Depression and dementia: cause, consequence or coincidence?. Maturitas. 2014;79(2):184‐190. doi:10.1016/j.maturitas.2014.05.009 24931304

[10] Byers AL , Yaffe K . Depression and risk of developing dementia. Nat Rev Neurol. 2011;7(6):323‐331. doi:10.1038/nrneurol.2011.60 21537355 PMC3327554

[11] Hayley S , Hakim AM , Albert PR . Depression, dementia and immune dysregulation. Brain. 2021;144(3):746‐760. doi:10.1093/brain/awaa405 33279966 PMC8041341

[12] Gerritsen L , Twait EL , Jonsson PV , Gudnason V , Launer LJ , Geerlings MI . Depression and dementia: the role of cortisol and vascular brain lesions. AGES‐Reykjavik Study. J Alzheimers Dis. 2022;85(4):1677‐1687. doi:10.3233/JAD-215241 34958034 PMC11044806

[13] Herrman H , Patel V , Kieling C , et al. Time for united action on depression: a Lancet‐World Psychiatric Association Commission. Lancet. 2022;399(10328):957‐1022. doi:10.1016/S0140-6736(21)02141-3 35180424

[14] Andresen EM , Malmgren JA , Carter WB , Patrick DL . Screening for depression in well older adults: evaluation of a short form of the CES‐D (Center for Epidemiologic Studies Depression Scale). Am J Prev Med. 1994;10(2):77‐84. 8037935

[15] Prince MJ , Reischies F , Beekman AT , et al. Development of the EURO‐D scale–a European, Union initiative to compare symptoms of depression in 14 European centres. Br J Psychiatry. 1999;174:330‐338. doi:10.1192/bjp.174.4.330 10533552

[16] Irwin M , Artin KH , Oxman MN . Screening for depression in the older adult: criterion validity of the 10‐item Center for Epidemiological Studies Depression Scale (CES‐D). Arch Intern Med. 1999;159(15):1701‐1704. doi:10.1001/archinte.159.15.1701 10448771

[17] Nam GE , Kim W , Han K , et al. Body weight variability and the risk of cardiovascular outcomes and mortality in patients with Type 2 diabetes: a nationwide cohort study. Diabetes Care. 2020;43(9):2234‐2241. doi:10.2337/dc19-2552 32641375

[18] Crimmins EM , Kim JK , Langa KM , Weir DR . Assessment of cognition using surveys and neuropsychological assessment: the Health and Retirement Study and the Aging, Demographics, and Memory Study. J Gerontol B Psychol Sci Soc Sci. 2011;66(Suppl 1):i162‐71. doi:10.1093/geronb/gbr048 21743047 PMC3165454

[19] Grasset L , Glymour MM , Yaffe K , et al. Association of traumatic brain injury with dementia and memory decline in older adults in the United States. Alzheimers Dement. 2020;16(6):853‐861. doi:10.1002/alz.12080 32323483 PMC7968111

[20] Ahmadi‐Abhari S , Guzman‐Castillo M , Bandosz P , et al. Temporal trend in dementia incidence since 2002 and projections for prevalence in England and Wales to 2040: modelling study. BMJ. 2017;358:j2856. doi:10.1136/bmj.j2856 28679494 PMC5497174

[21] Zheng F , Yan L , Yang Z , Zhong B , Xie W . HbA(1c), diabetes and cognitive decline: the English Longitudinal Study of Ageing. Diabetologia. 2018;61(4):839‐848. doi:10.1007/s00125-017-4541-7 29368156 PMC6448974

[22] Levine DA , Galecki AT , Langa KM , et al. Trajectory of cognitive decline after incident stroke. JAMA. 2015;314(1):41‐51. doi:10.1001/jama.2015.6968 26151265 PMC4655087

[23] Templ M , Alfons A , Filzmoser P . Exploring incomplete data using visualization techniques. Adv Data Anal Classificat. 2012;6(1):29‐47. doi:10.1007/s11634-011-0102-y

[24] van Buuren S , mice Groothuis‐OudshoornK . Multivariate imputation by chained equations in R. J Stat Softw. 2011;45(3):1‐67. doi:10.18637/jss.v045.i03

[25] Zhu Y , Li C , Wu T , et al. Associations of cumulative depressive symptoms with subsequent cognitive decline and adverse health events: two prospective cohort studies. J Affect Disord. 2023;320:91‐97. doi:10.1016/j.jad.2022.09.128 36183825

[26] Brain J , Alshahrani M , Kafadar AH , et al. Temporal dynamics in the association between depression and dementia: an umbrella review and meta‐analysis. EClinicalMedicine. 2025;84:103266. doi:10.1016/j.eclinm.2025.103266 40687743 PMC12273843

[27] Diniz BS , Butters MA , Albert SM , Dew MA , Reynolds CF . 3rd. Late‐life depression and risk of vascular dementia and Alzheimer's disease: systematic review and meta‐analysis of community‐based cohort studies. Br J Psychiatry. 2013;202(5):329‐335. doi:10.1192/bjp.bp.112.118307 23637108 PMC3640214

[28] Ouanes S , Popp J . High cortisol and the risk of dementia and Alzheimer's disease: a review of the literature. Front Aging Neurosci. 2019;11:43. doi:10.3389/fnagi.2019.00043 30881301 PMC6405479

[29] P S , Vellapandian C . Hypothalamic‐pituitary‐adrenal (HPA) axis: unveiling the potential mechanisms involved in stress‐induced Alzheimer's disease and depression. Cureus. 2024;16(8):e67595. doi:10.7759/cureus.67595 39310640 PMC11416836

[30] Linnemann C , Lang UE . Pathways connecting late‐life depression and dementia. Front Pharmacol. 2020;11:279. doi:10.3389/fphar.2020.00279 32231570 PMC7083108

[31] Zhao W , Zhao L , Chang X , Lu X , Tu Y . Elevated dementia risk, cognitive decline, and hippocampal atrophy in multisite chronic pain. Proc Natl Acad Sci U S A. 2023;120(9):e2215192120. doi:10.1073/pnas.2215192120 36802440 PMC9992778

[32] Xiao Y , Liao L , Huang K , Yao S , Gao L . Alzheimer's Disease Neuroimaging I. Coupling between hippocampal parenchymal fraction and cortical grey matter atrophy at different stages of cognitive decline. J Alzheimers Dis. 2023;93(2):791‐801. doi:10.3233/JAD-230124 37092228 PMC10200204

[33] Reuben DB , Kremen S , Maust DT . Dementia prevention and treatment: a narrative review. JAMA Intern Med. 2024;184(5):563‐572. doi:10.1001/jamainternmed.2023.8522 38436963

[34] Ferreira‐Vieira TH , Guimaraes IM , Silva FR , Ribeiro FM . Alzheimer's disease: targeting the Cholinergic System. Curr Neuropharmacol. 2016;14(1):101‐115. doi:10.2174/1570159x13666150716165726 26813123 PMC4787279

[35] Hampel H , Mesulam MM , Cuello AC , et al. The cholinergic system in the pathophysiology and treatment of Alzheimer's disease. Brain. 2018;141(7):1917‐1933. doi:10.1093/brain/awy132 29850777 PMC6022632

[36] Tian X , Chen S , Xu Q , et al. Magnitude and time course of insulin resistance accumulation with the risk of cardiovascular disease: an 11‐years cohort study. Cardiovasc Diabetol. 2023;22(1):339. doi:10.1186/s12933-023-02073-2 38093281 PMC10720129

[37] Liu Q , Cui H , Si F , Wu Y , Yu J . Association of cumulative exposure to metabolic score for visceral fat with the risk of cardiovascular disease and all‐cause mortality: a prospective cohort study. J Cachexia Sarcopenia Muscle. 2025;16(1):e13702. doi:10.1002/jcsm.13702 39935326 PMC11814533

