# Supplementary material for: Cumulative and variable depression symptom exposure and incident dementia: Panel data analysis of four longitudinal cohort studies
Source: Alzheimers Dement. 2025 Nov 28;21(11):e70950. doi: 10.1002/alz.70950 (PMC12662180; doi:10.1002/alz.70950)
Supplement: Supplementary file 2 — Supporting Information [file ALZ-21-e70950-s001.docx]

Supplementary Table 1 Cox regression models for the association between the cumulative exposure and incident dementia risk in ELSA

| **Categories** | | **Model 1** | | | **Model 2** | | | | | **Model 3** | | | | | **Model 4** | | | |  |
| --- | --- | --- | --- | --- | --- | --- | --- | --- | --- | --- | --- | --- | --- | --- | --- | --- | --- | --- | --- |
|  | | **HR** | **95% CI^1^** | **p-value** | **HR** | **95% CI** | | **p-value** | | **HR** | **95% CI** | | **p-value** | | **HR** | **95% CI** | | **p-value** | |
| **Cumulative CES-D** | | 1.07 | 1.05, 1.09 | **<0.001** | 1.07 | 1.04, 1.09 | | **<0.001** | | 1.06 | 1.04, 1.08 | | **<0.001** | | 1.06 | 1.04, 1.08 | | **<0.001** | |
| **Quartile cumulative CES-D** | |  |  |  |  |  | |  | |  |  | |  | |  |  | |  | |
| **Q1** | | Reference | | | Reference | | | | | Reference | | | | | Reference | | | | |
| **Q2** | | 1.03 | 0.56, 1.90 | 0.927 | 0.95 | 0.52, 1.76 | | 0.874 | | 0.94 | 0.51, 1.74 | | 0.841 | | 0.91 | 0.49, 1.68 | | 0.762 | |
| **Q3** | | 2.00 | 1.15, 3.49 | **0.014** | 1.63 | 0.92, 2.87 | | 0.093 | | 1.58 | 0.90, 2.79 | | 0.113 | | 1.60 | 0.90, 2.84 | | 0.107 | |
| **Q4** | | 3.65 | 2.21, 6.03 | **<0.001** | 2.96 | 1.73, 5.09 | | **<0.001** | | 2.74 | 1.59, 4.73 | | **<0.001** | | 2.71 | 1.56, 4.69 | | **<0.001** | |
| **P for trend** | |  |  | **<0.001** |  |  | | **<0.001** | |  |  | | **<0.001** | |  |  | | **<0.001** | |
| **Cumulative average CES-D** | | 1.30 | 1.22, 1.40 | **<0.001** | 1.29 | 1.18, 1.40 | | **<0.001** | | 1.26 | 1.16, 1.38 | | <0.001 | | 1.26 | 1.16, 1.38 | | **<0.001** | |
| **Quartile cumulative average CES-D** | |  |  |  |  |  | |  | |  |  | |  | |  |  | |  | |
| **Q1** | | Reference | | | Reference | | | | | Reference | | | | | Reference | | | | |
| **Q2** | | 1.03 | 0.56, 1.90 | 0.927 | 0.95 | 0.52, 1.76 | | 0.874 | | 0.94 | 0.51, 1.74 | | 0.841 | | 0.91 | 0.49, 1.68 | | 0.762 | |
| **Q3** | | 2.00 | 1.15, 3.49 | **0.014** | 1.63 | 0.92, 2.87 | | 0.093 | | 1.58 | 0.90, 2.79 | | 0.113 | | 1.60 | 0.90, 2.84 | | 0.107 | |
| **Q4** | | 3.65 | 2.21, 6.03 | **<0.001** | 2.96 | 1.73, 5.09 | | **<0.001** | | 2.74 | 1.59, 4.73 | | **<0.001** | | 2.71 | 1.56, 4.69 | | **<0.001** | |
| **P for trend** | |  |  | **<0.001** |  |  | | **<0.001** | |  |  | | **<0.001** | |  |  | | **<0.001** | |
| **Cumulative burden** | |  |  |  |  |  | |  | |  |  | |  | |  |  | |  | |
| **<0** | | Reference | | | Reference | | | | | Reference | | | | | Reference | | | | |
| **≥0** | | 2.93 | 2.09, 4.10 | **<0.001** | 2.55 | 1.78, 3.66 | | **<0.001** | | 2.37 | 1.64, 3.41 | | **<0.001** | | 2.36 | 1.62, 3.43 | | **<0.001** | |
| **Exposure duration** | |  |  |  |  |  | |  | |  |  | |  | |  |  | |  | |
| **0 year** | | Reference | | | Reference | | | | | Reference | | | | | Reference | | | | |
| **2 years** | | 1.99 | 1.15, 3.45 | **0.014** | 1.67 | 0.96, 2.92 | | 0.069 | | 1.48 | 0.84, 2.62 | | 0.175 | | 1.47 | 0.81, 2.66 | | 0.200 | |
| **4 years** | | 3.37 | 2.19, 5.18 | **<0.001** | 3.03 | 1.92, 4.78 | | **<0.001** | | 2.69 | 1.70, 4.27 | | **<0.001** | | 2.69 | 1.70, 4.25 | | **<0.001** | |
| **P for trend** | |  |  | **<0.001** |  |  | | **<0.001** | |  |  | | **<0.001** | |  |  | | **<0.001** | |
| **Slope** | |  |  |  |  |  | |  | |  |  | |  | |  |  | |  | |
| **<0** | | Reference | | | Reference | | | | | Reference | | | | | Reference | | | | |
| **≥0** | | 0.79 | 0.56, 1.09 | 0.152 | 0.74 | 0.53, 1.03 | | 0.073 | | 0.78 | 0.56, 1.09 | | 0.144 | | 0.77 | 0.55, 1.08 | | 0.129 | |
| **Time course patterns** | |  |  |  |  |  | |  | |  |  | |  | |  |  | |  | |
| **Decrease-decrease** | | Reference | | | Reference | | | | | Reference | | | | | Reference | | | | |
| **Decrease-increase** | | 1.65 | 1.09, 2.50 | **0.019** | 1.37 | 0.90, 2.10 | | 0.144 | | 1.37 | 0.90, 2.10 | | 0.146 | | 1.40 | 0.92, 2.15 | | 0.119 | |
| **Increase-decrease** | | 1.63 | 1.07, 2.46 | **0.022** | 1.39 | 0.91, 2.11 | | 0.123 | | 1.40 | 0.92, 2.12 | | 0.115 | | 1.43 | 0.93, 2.19 | | 0.099 | |
| **Increase-increase** | | 2.36 | 1.21, 4.63 | **0.012** | 1.59 | 0.79, 3.20 | | 0.190 | | 1.50 | 0.75, 3.01 | | 0.250 | | 1.37 | 0.66, 2.85 | | 0.396 | |
| CI^1^ = Confidence Interval, HR = Hazard Ratio | | | |  |  |  | |  | |  |  | |  | |  |  | |  |  |

Model 1: unadjusted

Model 2: adjusted for age, gender, marital status, education level

Model 3: adjusted for Model 2 plus smoking status, drinking status, HbA1c, TC, HDL-c, CRP

Model 4: adjusted for Model 3 plus depression, hypertension, diabetes, heart disease, stroke

Supplementary Table 2 Cox regression models for the association between the cumulative exposure and incident dementia risk in HRS

| **Categories** | | **Model 1** | | | **Model 2** | | | | | **Model 3** | | | | | **Model 4** | | | |  |
| --- | --- | --- | --- | --- | --- | --- | --- | --- | --- | --- | --- | --- | --- | --- | --- | --- | --- | --- | --- |
|  | | **HR** | **95% CI^1^** | **p-value** | **HR** | **95% CI** | | **p-value** | | **HR** | **95% CI** | | **p-value** | | **HR** | **95% CI** | | **p-value** | |
| **Cumulative CES-D** | | 1.08 | 1.06, 1.10 | **<0.001** | 1.09 | 1.07, 1.11 | | **<0.001** | | 1.09 | 1.07, 1.11 | | **<0.001** | | 1.08 | 1.07, 1.10 | | **<0.001** | |
| **Quartile cumulative CES-D** | |  |  |  |  |  | |  | |  |  | |  | |  |  | |  | |
| **Q1** | | Reference | | | Reference | | | | | Reference | | | | | Reference | | | | |
| **Q2** | | 4.46 | 1.51, 13.2 | **0.007** | 4.11 | 1.39, 12.2 | | **0.011** | | 4.18 | 1.41, 12.4 | | **0.010** | | 4.07 | 1.37, 12.1 | | **0.012** | |
| **Q3** | | 10.5 | 3.77, 29.2 | **<0.001** | 10.2 | 3.62, 28.9 | | **<0.001** | | 10.1 | 3.55, 28.6 | | **<0.001** | | 9.63 | 3.36, 27.6 | | **<0.001** | |
| **Q4** | | 18.5 | 6.75, 50.7 | **<0.001** | 20.7 | 7.41, 58.0 | | **<0.001** | | 20.4 | 7.28, 57.2 | | **<0.001** | | 18.0 | 6.35, 51.0 | | **<0.001** | |
| **P for trend** | |  |  | **<0.001** |  |  | | **<0.001** | |  |  | | **<0.001** | |  |  | | **<0.001** | |
| **Cumulative average CES-D** | | 1.36 | 1.28, 1.44 | **<0.001** | 1.43 | 1.33, 1.53 | | **<0.001** | | 1.42 | 1.32, 1.52 | | **<0.001** | | 1.39 | 1.29, 1.49 | | **<0.001** | |
| **Quartile cumulative average CES-D** | |  |  |  |  |  | |  | |  |  | |  | |  |  | |  | |
| **Q1** | | Reference | | | Reference | | | | | Reference | | | | | Reference | | | | |
| **Q2** | | 4.46 | 1.51, 13.2 | **0.007** | 4.11 | 1.39, 12.2 | | **0.011** | | 4.18 | 1.41, 12.4 | | **0.010** | | 4.07 | 1.37, 12.1 | | **0.012** | |
| **Q3** | | 10.5 | 3.77, 29.2 | **<0.001** | 10.2 | 3.62, 28.9 | | **<0.001** | | 10.1 | 3.55, 28.6 | | **<0.001** | | 9.63 | 3.36, 27.6 | | **<0.001** | |
| **Q4** | | 18.5 | 6.75, 50.7 | **<0.001** | 20.7 | 7.41, 58.0 | | **<0.001** | | 20.4 | 7.28, 57.2 | | **<0.001** | | 18.0 | 6.35, 51.0 | | **<0.001** | |
| **P for trend** | |  |  | **<0.001** |  |  | | **<0.001** | |  |  | | **<0.001** | |  |  | | **<0.001** | |
| **Cumulative burden** | |  |  |  |  |  | |  | |  |  | |  | |  |  | |  | |
| **<0** | | Reference | | | Reference | | | | | Reference | | | | | Reference | | | | |
| **≥0** | | 5.46 | 3.58, 8.34 | **<0.001** | 5.76 | 3.72, 8.91 | | **<0.001** | | 5.57 | 3.59, 8.64 | | **<0.001** | | 5.10 | 3.26, 7.98 | | **<0.001** | |
| **Exposure duration** | |  |  |  |  |  | |  | |  |  | |  | |  |  | |  | |
| **0 year** | | Reference | | | Reference | | | | | Reference | | | | | Reference | | | | |
| **2 years** | | 3.17 | 2.00, 5.04 | **<0.001** | 3.21 | 1.98, 5.22 | | **<0.001** | | 3.17 | 1.96, 5.14 | | **<0.001** | | 2.88 | 1.77, 4.69 | | **<0.001** | |
| **4 years** | | 3.84 | 2.61, 5.65 | **<0.001** | 4.19 | 2.80, 6.28 | | **<0.001** | | 4.06 | 2.72, 6.06 | | **<0.001** | | 3.60 | 2.38, 5.42 | | **<0.001** | |
| **P for trend** | |  |  | **<0.001** |  |  | | **<0.001** | |  |  | | **<0.001** | |  |  | | **<0.001** | |
| **Slope** | |  |  |  |  |  | |  | |  |  | |  | |  |  | |  | |
| **<0** | | Reference | | | Reference | | | | | Reference | | | | | Reference | | | | |
| **≥0** | | 0.71 | 0.51, 1.01 | 0.054 | 0.70 | 0.49, 1.00 | | **0.049** | | 0.68 | 0.48, 0.97 | | **0.034** | | 0.72 | 0.51, 1.04 | | 0.079 | |
| **Time course patterns** | |  |  |  |  |  | |  | |  |  | |  | |  |  | |  | |
| **Decrease-decrease** | | Reference | | | Reference | | | | | Reference | | | | | Reference | | | | |
| **Decrease-increase** | | 3.50 | 2.21, 5.52 | **<0.001** | 3.04 | 1.91, 4.82 | | **<0.001** | | 2.95 | 1.84, 4.71 | | **<0.001** | | 2.94 | 1.84, 4.69 | | **<0.001** | |
| **Increase-decrease** | | 2.85 | 1.78, 4.57 | **<0.001** | 2.67 | 1.66, 4.29 | | **<0.001** | | 2.59 | 1.60, 4.20 | | **<0.001** | | 2.50 | 1.55, 4.04 | | **<0.001** | |
| **Increase-increase** | | 4.67 | 2.46, 8.88 | **<0.001** | 3.87 | 1.99, 7.51 | | **<0.001** | | 3.61 | 1.86, 7.01 | | **<0.001** | | 3.27 | 1.70, 6.28 | | **<0.001** | |
| CI^1^ = Confidence Interval, HR = Hazard Ratio | | | |  |  |  | |  | |  |  | |  | |  |  | |  |  |

Model 1: unadjusted

Model 2: adjusted for age, gender, marital status, education level, residence

Model 3: adjusted for Model 2 plus smoking status, drinking status, HbA1c, TC, HDL-c, CRP

Model 4: adjusted for Model 3 plus depression, hypertension, diabetes, heart disease, stroke

Supplementary Table 3 Cox regression models for the association between the cumulative exposure and incident dementia risk in SHARE

| **Categories** | | **Model 1** | | | **Model 2** | | | | | **Model 3** | | | | | **Model 4** | | | |  |
| --- | --- | --- | --- | --- | --- | --- | --- | --- | --- | --- | --- | --- | --- | --- | --- | --- | --- | --- | --- |
|  | | **HR** | **95% CI^1^** | **p-value** | **HR** | **95% CI** | | **p-value** | | **HR** | **95% CI** | | **p-value** | | **HR** | **95% CI** | | **p-value** | |
| **Cumulative EURO-D** | | 1.09 | 1.08, 1.11 | **<0.001** | 1.08 | 1.06, 1.10 | | **<0.001** | | 1.08 | 1.06, 1.10 | | **<0.001** | | 1.08 | 1.06, 1.09 | | **<0.001** | |
| **Quartile cumulative EURO-D** | |  |  |  |  |  | |  | |  |  | |  | |  |  | |  | |
| **Q1** | | Reference | | | Reference | | | | | Reference | | | | | Reference | | | | |
| **Q2** | | 3.11 | 1.46, 6.62 | **0.003** | 2.81 | 1.32, 5.97 | | **0.007** | | 2.84 | 1.33, 6.05 | | **0.007** | | 2.66 | 1.25, 5.68 | | **0.011** | |
| **Q3** | | 6.35 | 3.15, 12.8 | **<0.001** | 5.28 | 2.60, 10.7 | | **<0.001** | | 5.23 | 2.58, 10.6 | | **<0.001** | | 4.72 | 2.32, 9.61 | | **<0.001** | |
| **Q4** | | 12.2 | 6.18, 24.2 | **<0.001** | 8.74 | 4.35, 17.5 | | **<0.001** | | 8.54 | 4.25, 17.2 | | **<0.001** | | 7.35 | 3.64, 14.8 | | **<0.001** | |
| **P for trend** | |  |  | **<0.001** |  |  | | **<0.001** | |  |  | | **<0.001** | |  |  | | **<0.001** | |
| **Cumulative average EURO-D** | | 1.42 | 1.34, 1.50 | **<0.001** | 1.37 | 1.28, 1.46 | | **<0.001** | | 1.37 | 1.28, 1.47 | | **<0.001** | | 1.34 | 1.25, 1.44 | | **<0.001** | |
| **Quartile cumulative average EURO-D** | |  |  |  |  |  | |  | |  |  | |  | |  |  | |  | |
| **Q1** | | Reference | | | Reference | | | | | Reference | | | | | Reference | | | | |
| **Q2** | | 3.11 | 1.46, 6.62 | **0.003** | 2.81 | 1.32, 5.97 | | **0.007** | | 2.84 | 1.33, 6.05 | | **0.007** | | 2.66 | 1.25, 5.68 | | **0.011** | |
| **Q3** | | 6.35 | 3.15, 12.8 | **<0.001** | 5.28 | 2.60, 10.7 | | **<0.001** | | 5.23 | 2.58, 10.6 | | **<0.001** | | 4.72 | 2.32, 9.61 | | **<0.001** | |
| **Q4** | | 12.2 | 6.18, 24.2 | **<0.001** | 8.74 | 4.35, 17.5 | | **<0.001** | | 8.54 | 4.25, 17.2 | | **<0.001** | | 7.35 | 3.64, 14.8 | | **<0.001** | |
| **P for trend** | |  |  | **<0.001** |  |  | | **<0.001** | |  |  | | **<0.001** | |  |  | | **<0.001** | |
| **Cumulative burden** | |  |  |  |  |  | |  | |  |  | |  | |  |  | |  | |
| **<0** | | Reference | | | Reference | | | | | Reference | | | | | Reference | | | | |
| **≥0** | | 4.15 | 3.10, 5.57 | **<0.001** | 3.14 | 2.28, 4.34 | | **<0.001** | | 3.08 | 2.23, 4.25 | | **<0.001** | | 2.80 | 2.02, 3.88 | | **<0.001** | |
| **Exposure duration** | |  |  |  |  |  | |  | |  |  | |  | |  |  | |  | |
| **0 year** | | Reference | | | Reference | | | | | Reference | | | | | Reference | | | | |
| **2 years** | | 2.71 | 1.74, 4.22 | **<0.001** | 2.18 | 1.37, 3.47 | | **0.001** | | 2.12 | 1.33, 3.39 | | **0.002** | | 1.98 | 1.24, 3.15 | | **0.004** | |
| **4 years** | | 5.47 | 3.82, 7.82 | **<0.001** | 4.32 | 2.93, 6.39 | | **<0.001** | | 4.33 | 2.93, 6.42 | | **<0.001** | | 3.89 | 2.61, 5.78 | | **<0.001** | |
| **P for trend** | |  |  | **<0.001** |  |  | | **<0.001** | |  |  | | **<0.001** | |  |  | | **<0.001** | |
| **Slope** | |  |  |  |  |  | |  | |  |  | |  | |  |  | |  | |
| **<0** | | Reference | | | Reference | | | | | Reference | | | | | Reference | | | | |
| **≥0** | | 1.24 | 0.94, 1.65 | 0.130 | 1.13 | 0.85, 1.50 | | 0.400 | | 1.13 | 0.85, 1.50 | | 0.397 | | 1.16 | 0.87, 1.55 | | 0.299 | |
| **Time course patterns** | |  |  |  |  |  | |  | |  |  | |  | |  |  | |  | |
| **Decrease-decrease** | | Reference | | | Reference | | | | | Reference | | | | | Reference | | | | |
| **Decrease-increase** | | 2.07 | 1.39, 3.06 | **<0.001** | 1.72 | 1.16, 2.56 | | **0.007** | | 1.71 | 1.15, 2.54 | | **0.008** | | 1.67 | 1.12, 2.48 | | **0.012** | |
| **Increase-decrease** | | 1.76 | 1.18, 2.64 | **0.006** | 1.38 | 0.92, 2.07 | | 0.120 | | 1.37 | 0.91, 2.06 | | 0.134 | | 1.36 | 0.90, 2.05 | | 0.141 | |
| **Increase-increase** | | 2.41 | 1.45, 4.03 | **<0.001** | 1.54 | 0.92, 2.59 | | 0.101 | | 1.60 | 0.95, 2.69 | | 0.074 | | 1.41 | 0.83, 2.37 | | 0.201 | |
| CI^1^ = Confidence Interval, HR = Hazard Ratio | | | |  |  |  | |  | |  |  | |  | |  |  | |  |  |

Model 1: unadjusted

Model 2: adjusted for age, gender, marital status, education level, residence

Model 3: adjusted for Model 2 plus smoking status, drinking status, HbA1c, TC, HDL-c, CRP

Model 4: adjusted for Model 3 plus depression, hypertension, diabetes, heart disease, stroke

Supplementary Table 4 Cox regression models for the association between the cumulative exposure and incident dementia risk in CHARLS

| **Categories** | | **Model 1** | | | **Model 2** | | | | | **Model 3** | | | | | **Model 4** | | | |  |
| --- | --- | --- | --- | --- | --- | --- | --- | --- | --- | --- | --- | --- | --- | --- | --- | --- | --- | --- | --- |
|  | | **HR** | **95% CI^1^** | **p-value** | **HR** | **95% CI** | | **p-value** | | **HR** | **95% CI** | | **p-value** | | **HR** | **95% CI** | | **p-value** | |
| **Cumulative CES-D** | | 1.03 | 1.03, 1.04 | **<0.001** | 1.04 | 1.03, 1.04 | | **<0.001** | | 1.04 | 1.03, 1.04 | | **<0.001** | | 1.03 | 1.03, 1.04 | | **<0.001** | |
| **Quartile cumulative CES-D** | |  |  |  |  |  | |  | |  |  | |  | |  |  | |  | |
| **Q1** | | Reference | | | Reference | | | | | Reference | | | | | Reference | | | | |
| **Q2** | | 1.77 | 0.99, 3.15 | 0.054 | 1.63 | 0.91, 2.91 | | 0.099 | | 1.62 | 0.90, 2.89 | | 0.106 | | 1.52 | 0.85, 2.72 | | 0.161 | |
| **Q3** | | 2.99 | 1.76, 5.07 | **<0.001** | 2.86 | 1.68, 4.89 | | **<0.001** | | 2.85 | 1.67, 4.87 | | **<0.001** | | 2.70 | 1.58, 4.63 | | **<0.001** | |
| **Q4** | | 7.54 | 4.64, 12.3 | **<0.001** | 7.86 | 4.78, 12.9 | | **<0.001** | | 7.61 | 4.62, 12.5 | | **<0.001** | | 7.07 | 4.27, 11.7 | | **<0.001** | |
| **P for trend** | |  |  | **<0.001** |  |  | | **<0.001** | |  |  | | **<0.001** | |  |  | | **<0.001** | |
| **Cumulative average CES-D** | | 1.14 | 1.12, 1.17 | **<0.001** | 1.15 | 1.13, 1.18 | | **<0.001** | | 1.15 | 1.13, 1.18 | | **<0.001** | | 1.15 | 1.12, 1.17 | | **<0.001** | |
| **Quartile cumulative average CES-D** | |  |  |  |  |  | |  | |  |  | |  | |  |  | |  | |
| **Q1** | | Reference | | | Reference | | | | | Reference | | | | | Reference | | | | |
| **Q2** | | 1.77 | 0.99, 3.15 | 0.054 | 1.63 | 0.91, 2.91 | | 0.099 | | 1.62 | 0.90, 2.89 | | 0.106 | | 1.52 | 0.85, 2.72 | | 0.161 | |
| **Q3** | | 2.99 | 1.76, 5.07 | **<0.001** | 2.86 | 1.68, 4.89 | | **<0.001** | | 2.85 | 1.67, 4.87 | | **<0.001** | | 2.70 | 1.58, 4.63 | | **<0.001** | |
| **Q4** | | 7.54 | 4.64, 12.3 | **<0.001** | 7.86 | 4.78, 12.9 | | **<0.001** | | 7.61 | 4.62, 12.5 | | **<0.001** | | 7.07 | 4.27, 11.7 | | **<0.001** | |
| **P for trend** | |  |  | **<0.001** |  |  | | **<0.001** | |  |  | | **<0.001** | |  |  | | **<0.001** | |
| **Cumulative burden** | |  |  |  |  |  | |  | |  |  | |  | |  |  | |  | |
| **<0** | | Reference | | | Reference | | | | | Reference | | | | | Reference | | | | |
| **≥0** | | 3.77 | 2.71, 5.24 | **<0.001** | 3.74 | 2.68, 5.23 | | **<0.001** | | 3.69 | 2.63, 5.17 | | **<0.001** | | 3.55 | 2.53, 4.99 | | **<0.001** | |
| **Exposure duration** | |  |  |  |  |  | |  | |  |  | |  | |  |  | |  | |
| **0 year** | | Reference | | | Reference | | | | | Reference | | | | | Reference | | | | |
| **2 years** | | 2.05 | 1.38, 3.06 | **<0.001** | 2.00 | 1.34, 2.99 | | **<0.001** | | 1.95 | 1.31, 2.92 | | **0.001** | | 1.92 | 1.28, 2.88 | | **0.001** | |
| **4 years** | | 3.94 | 2.91, 5.34 | **<0.001** | 4.12 | 3.02, 5.62 | | **<0.001** | | 4.00 | 2.92, 5.47 | | **<0.001** | | 3.82 | 2.79, 5.25 | | **<0.001** | |
| **P for trend** | |  |  | **<0.001** |  |  | | **<0.001** | |  |  | | **<0.001** | |  |  | | **<0.001** | |
| **Slope** | |  |  |  |  |  | |  | |  |  | |  | |  |  | |  | |
| **<0** | | Reference | | | Reference | | | | | Reference | | | | | Reference | | | | |
| **≥0** | | 1.29 | 0.98, 1.68 | 0.069 | 1.30 | 0.99, 1.71 | | 0.057 | | 1.31 | 1.0, 1.71 | | 0.054 | | 1.37 | 1.04, 1.80 | | **0.023** | |
| **Time course patterns** | |  |  |  |  |  | |  | |  |  | |  | |  |  | |  | |
| **Decrease-decrease** | | Reference | | | Reference | | | | | Reference | | | | | Reference | | | | |
| **Decrease-increase** | | 2.11 | 1.40, 3.20 | **<0.001** | 2.18 | 1.44, 3.30 | | **<0.001** | | 2.21 | 1.46, 3.35 | | **<0.001** | | 2.22 | 1.46, 3.36 | | **<0.001** | |
| **Increase-decrease** | | 1.79 | 1.17, 2.74 | **0.007** | 1.92 | 1.26, 2.95 | | **0.003** | | 1.95 | 1.27, 2.98 | | **0.002** | | 2.06 | 1.34, 3.16 | | **<0.001** | |
| **Increase-increase** | | 1.91 | 1.16, 3.15 | **0.011** | 1.91 | 1.16, 3.14 | | **0.011** | | 1.90 | 1.15, 3.13 | | **0.012** | | 1.99 | 1.20, 3.28 | | **0.007** | |
| CI^1^ = Confidence Interval, HR = Hazard Ratio | | | |  |  |  | |  | |  |  | |  | |  |  | |  |  |

Model 1: unadjusted

Model 2: adjusted for age, gender, marital status, education level, residence

Model 3: adjusted for Model 2 plus smoking status, drinking status, HbA1c, TC, HDL-c, CRP

Model 4: adjusted for Model 3 plus depression, hypertension, diabetes, heart disease, stroke

Supplementary Table 5 Cox regression models for the association between the cumulative exposure and incident dementia risk in ELSA with lagged analyses

| **Categories** | | **Model 1** | | | **Model 2** | | | | | **Model 3** | | | | | **Model 4** | | | |  |
| --- | --- | --- | --- | --- | --- | --- | --- | --- | --- | --- | --- | --- | --- | --- | --- | --- | --- | --- | --- |
|  | | **HR** | **95% CI^1^** | **p-value** | **HR** | **95% CI** | | **p-value** | | **HR** | **95% CI** | | **p-value** | | **HR** | **95% CI** | | **p-value** | |
| **Cumulative CES-D** | | 1.07 | 1.05, 1.09 | **<0.001** | 1.07 | 1.04, 1.09 | | **<0.001** | | 1.06 | 1.04, 1.09 | | **<0.001** | | 1.06 | 1.04, 1.09 | | **<0.001** | |
| **Quartile cumulative CES-D** | |  |  |  |  |  | |  | |  |  | |  | |  |  | |  | |
| **Q1** | | Reference | | | Reference | | | | | Reference | | | | | Reference | | | | |
| **Q2** | | 1.30 | 0.66, 2.55 | 0.441 | 1.21 | 0.62, 2.36 | | 0.582 | | 1.19 | 0.61, 2.33 | | 0.618 | | 1.16 | 0.59, 2.27 | | 0.670 | |
| **Q3** | | 2.42 | 1.30, 4.49 | **0.005** | 1.98 | 1.05, 3.72 | | **0.035** | | 1.92 | 1.02, 3.61 | | **0.043** | | 1.94 | 1.02, 3.67 | | **0.042** | |
| **Q4** | | 4.18 | 2.36, 7.40 | **<0.001** | 3.50 | 1.90, 6.42 | | **<0.001** | | 3.21 | 1.73, 5.95 | | **<0.001** | | 3.24 | 1.73, 6.05 | | **<0.001** | |
| **P for trend** | |  |  | **<0.001** |  |  | | **<0.001** | |  |  | | **<0.001** | |  |  | | **<0.001** | |
| **Cumulative average CES-D** | | 1.30 | 1.21, 1.40 | **<0.001** | 1.30 | 1.18, 1.42 | | **<0.001** | | 1.28 | 1.16, 1.40 | | **<0.001** | | 1.28 | 1.16, 1.42 | | **<0.001** | |
| **Quartile cumulative average CES-D** | |  |  |  |  |  | |  | |  |  | |  | |  |  | |  | |
| **Q1** | | Reference | | | Reference | | | | | Reference | | | | | Reference | | | | |
| **Q2** | | 1.30 | 0.66, 2.55 | 0.441 | 1.21 | 0.62, 2.36 | | 0.582 | | 1.19 | 0.61, 2.33 | | 0.618 | | 1.16 | 0.59, 2.27 | | 0.670 | |
| **Q3** | | 2.42 | 1.30, 4.49 | **0.005** | 1.98 | 1.05, 3.72 | | **0.035** | | 1.92 | 1.02, 3.61 | | **0.043** | | 1.94 | 1.02, 3.67 | | **0.042** | |
| **Q4** | | 4.18 | 2.36, 7.40 | **<0.001** | 3.50 | 1.90, 6.42 | | **<0.001** | | 3.21 | 1.73, 5.95 | | **<0.001** | | 3.24 | 1.73, 6.05 | | **<0.001** | |
| **P for trend** | |  |  | **<0.001** |  |  | | **<0.001** | |  |  | | **<0.001** | |  |  | | **<0.001** | |
| **Cumulative burden** | |  |  |  |  |  | |  | |  |  | |  | |  |  | |  | |
| **<0** | | Reference | | | Reference | | | | | Reference | | | | | Reference | | | | |
| **≥0** | | 2.90 | 2.02, 4.17 | **<0.001** | 2.59 | 1.76, 3.80 | | **<0.001** | | 2.40 | 1.62, 3.55 | | **<0.001** | | 2.44 | 1.63, 3.64 | | **<0.001** | |
| **Exposure duration** | |  |  |  |  |  | |  | |  |  | |  | |  |  | |  | |
| **0 year** | | Reference | | | Reference | | | | | Reference | | | | | Reference | | | | |
| **2 years** | | 2.17 | 1.22, 3.84 | **0.008** | 1.85 | 1.04, 3.29 | | **0.035** | | 1.65 | 0.91, 3.00 | | 0.100 | | 1.68 | 0.91, 3.10 | | 0.097 | |
| **4 years** | | 3.36 | 2.10, 5.37 | **<0.001** | 3.20 | 1.92, 5.33 | | **<0.001** | | 2.82 | 1.69, 4.72 | | **<0.001** | | 2.85 | 1.71, 4.74 | | **<0.001** | |
| **P for trend** | |  |  | **<0.001** |  |  | | **<0.001** | |  |  | | **<0.001** | |  |  | | **<0.001** | |
| **Slope** | |  |  |  |  |  | |  | |  |  | |  | |  |  | |  | |
| **<0** | | Reference | | | Reference | | | | | Reference | | | | | Reference | | | | |
| **≥0** | | 0.77 | 0.54, 1.09 | 0.142 | 0.72 | 0.50, 1.02 | | 0.065 | | 0.75 | 0.52, 1.07 | | 0.114 | | 0.73 | 0.51, 1.05 | | 0.092 | |
| **Time course patterns** | |  |  |  |  |  | |  | |  |  | |  | |  |  | |  | |
| **Decrease-decrease** | | Reference | | | Reference | | | | | Reference | | | | | Reference | | | | |
| **Decrease-increase** | | 1.71 | 1.10, 2.66 | **0.018** | 1.43 | 0.91, 2.25 | | 0.124 | | 1.42 | 0.91, 2.23 | | 0.126 | | 1.46 | 0.93, 2.29 | | 0.102 | |
| **Increase-decrease** | | 1.55 | 0.99, 2.44 | 0.058 | 1.33 | 0.84, 2.10 | | 0.218 | | 1.34 | 0.85, 2.11 | | 0.206 | | 1.38 | 0.87, 2.19 | | 0.175 | |
| **Increase-increase** | | 2.48 | 1.22, 5.05 | **0.012** | 1.69 | 0.81, 3.50 | | 0.160 | | 1.54 | 0.74, 3.22 | | 0.251 | | 1.43 | 0.65, 3.12 | | 0.373 | |
| CI^1^ = Confidence Interval, HR = Hazard Ratio | | | |  |  |  | |  | |  |  | |  | |  |  | |  |  |

Model 1: unadjusted

Model 2: adjusted for age, gender, marital status, education level

Model 3: adjusted for Model 2 plus smoking status, drinking status, HbA1c, TC, HDL-c, CRP

Model 4: adjusted for Model 3 plus depression, hypertension, diabetes, heart disease, stroke

Supplementary Table 6 Cox regression models for the association between the cumulative exposure and incident dementia risk in HRS with lagged analyses

| **Categories** | | **Model 1** | | | **Model 2** | | | | | **Model 3** | | | | | **Model 4** | | | |  |
| --- | --- | --- | --- | --- | --- | --- | --- | --- | --- | --- | --- | --- | --- | --- | --- | --- | --- | --- | --- |
|  | | **HR** | **95% CI^1^** | **p-value** | **HR** | **95% CI** | | **p-value** | | **HR** | **95% CI** | | **p-value** | | **HR** | **95% CI** | | **p-value** | |
| **Cumulative CES-D** | | 1.08 | 1.06, 1.10 | **<0.001** | 1.10 | 1.08, 1.12 | | **<0.001** | | 1.10 | 1.08, 1.12 | | **<0.001** | | 1.09 | 1.07, 1.11 | | **<0.001** | |
| **Quartile cumulative CES-D** | |  |  |  |  |  | |  | |  |  | |  | |  |  | |  | |
| **Q1** | | Reference | | | Reference | | | | | Reference | | | | | Reference | | | | |
| **Q2** | | 4.71 | 1.35, 16.4 | **0.015** | 4.50 | 1.29, 15.7 | | **0.018** | | 4.67 | 1.34, 16.3 | | **0.016** | | 4.50 | 1.29, 15.7 | | **0.018** | |
| **Q3** | | 7.90 | 2.36, 26.4 | **<0.001** | 8.04 | 2.39, 27.0 | | **<0.001** | | 8.19 | 2.42, 27.7 | | **<0.001** | | 7.81 | 2.29, 26.6 | | **0.001** | |
| **Q4** | | 18.8 | 5.89, 60.1 | **<0.001** | 21.5 | 6.61, 70.1 | | **<0.001** | | 22.3 | 6.85, 72.9 | | **<0.001** | | 18.9 | 5.79, 62.0 | | **<0.001** | |
| **P for trend** | |  |  | **<0.001** |  |  | | **<0.001** | |  |  | | **<0.001** | |  |  | | **<0.001** | |
| **Cumulative average CES-D** | | 1.37 | 1.28, 1.48 | **<0.001** | 1.45 | 1.34, 1.58 | | **<0.001** | | 1.45 | 1.34, 1.58 | | **<0.001** | | 1.41 | 1.30, 1.54 | | **<0.001** | |
| **Quartile cumulative average CES-D** | |  |  |  |  |  | |  | |  |  | |  | |  |  | |  | |
| **Q1** | | Reference | | | Reference | | | | | Reference | | | | | Reference | | | | |
| **Q2** | | 4.71 | 1.35, 16.4 | **0.015** | 4.50 | 1.29, 15.7 | | **0.018** | | 4.67 | 1.34, 16.3 | | **0.016** | | 4.50 | 1.29, 15.7 | | **0.018** | |
| **Q3** | | 7.90 | 2.36, 26.4 | **<0.001** | 8.04 | 2.39, 27.0 | | **<0.001** | | 8.19 | 2.42, 27.7 | | **<0.001** | | 7.81 | 2.29, 26.6 | | **0.001** | |
| **Q4** | | 18.8 | 5.89, 60.1 | **<0.001** | 21.5 | 6.61, 70.1 | | **<0.001** | | 22.3 | 6.85, 72.9 | | **<0.001** | | 18.9 | 5.79, 62.0 | | **<0.001** | |
| **P for trend** | |  |  | **<0.001** |  |  | | **<0.001** | |  |  | | **<0.001** | |  |  | | **<0.001** | |
| **Cumulative burden** | |  |  |  |  |  | |  | |  |  | |  | |  |  | |  | |
| **<0** | | Reference | | | Reference | | | | | Reference | | | | | Reference | | | | |
| **≥0** | | 5.26 | 3.20, 8.63 | **<0.001** | 5.68 | 3.41, 9.47 | | **<0.001** | | 5.67 | 3.41, 9.44 | | **<0.001** | | 5.07 | 3.02, 8.53 | | **<0.001** | |
| **Exposure duration** | |  |  |  |  |  | |  | |  |  | |  | |  |  | |  | |
| **0 year** | | Reference | | | Reference | | | | | Reference | | | | | Reference | | | | |
| **2 years** | | 3.56 | 2.05, 6.19 | **<0.001** | 3.80 | 2.15, 6.71 | | **<0.001** | | 3.82 | 2.17, 6.73 | | **<0.001** | | 3.41 | 1.92, 6.07 | | **<0.001** | |
| **4 years** | | 4.44 | 2.80, 7.04 | **<0.001** | 5.09 | 3.17, 8.18 | | **<0.001** | | 5.07 | 3.19, 8.07 | | **<0.001** | | 4.37 | 2.70, 7.06 | | **<0.001** | |
| **P for trend** | |  |  | **<0.001** |  |  | | **<0.001** | |  |  | | **<0.001** | |  |  | | **<0.001** | |
| **Slope** | |  |  |  |  |  | |  | |  |  | |  | |  |  | |  | |
| **<0** | | Reference | | | Reference | | | | | Reference | | | | | Reference | | | | |
| **≥0** | | 0.62 | 0.41, 0.93 | **0.022** | 0.60 | 0.40, 0.91 | | **0.017** | | 0.58 | 0.38, 0.89 | | **0.013** | | 0.63 | 0.41, 0.96 | | **0.032** | |
| **Time course patterns** | |  |  |  |  |  | |  | |  |  | |  | |  |  | |  | |
| **Decrease-decrease** | | Reference | | | Reference | | | | | Reference | | | | | Reference | | | | |
| **Decrease-increase** | | 3.36 | 1.93, 5.84 | **<0.001** | 3.00 | 1.72, 5.24 | | **<0.001** | | 2.97 | 1.68, 5.26 | | **<0.001** | | 2.85 | 1.60, 5.07 | | **<0.001** | |
| **Increase-decrease** | | 2.99 | 1.71, 5.24 | **<0.001** | 2.84 | 1.61, 5.00 | | **<0.001** | | 2.85 | 1.60, 5.06 | | **<0.001** | | 2.68 | 1.51, 4.78 | | **<0.001** | |
| **Increase-increase** | | 5.20 | 2.47, 10.9 | **<0.001** | 4.54 | 2.08, 9.88 | | **<0.001** | | 4.35 | 2.00, 9.46 | | **<0.001** | | 3.93 | 1.85, 8.38 | | **<0.001** | |
| CI^1^ = Confidence Interval, HR = Hazard Ratio | | | |  |  |  | |  | |  |  | |  | |  |  | |  |  |

Model 1: unadjusted

Model 2: adjusted for age, gender, marital status, education level

Model 3: adjusted for Model 2 plus smoking status, drinking status, HbA1c, TC, HDL-c, CRP

Model 4: adjusted for Model 3 plus depression, hypertension, diabetes, heart disease, stroke

Supplementary Table 7 Cox regression models for the association between the cumulative exposure and incident dementia risk in SHARE with lagged analyses

| **Categories** | | **Model 1** | | | **Model 2** | | | | | **Model 3** | | | | | **Model 4** | | | |  |
| --- | --- | --- | --- | --- | --- | --- | --- | --- | --- | --- | --- | --- | --- | --- | --- | --- | --- | --- | --- |
|  | | **HR** | **95% CI^1^** | **p-value** | **HR** | **95% CI** | | **p-value** | | **HR** | **95% CI** | | **p-value** | | **HR** | **95% CI** | | **p-value** | |
| **Cumulative EURO-D** | | 1.09 | 1.07, 1.10 | **<0.001** | 1.08 | 1.06, 1.10 | | **<0.001** | | 1.08 | 1.06, 1.10 | | **<0.001** | | 1.08 | 1.06, 1.09 | | **<0.001** | |
| **Quartile cumulative EURO-D** | |  |  |  |  |  | |  | |  |  | |  | |  |  | |  | |
| **Q1** | | Reference | | | Reference | | | | | Reference | | | | | Reference | | | | |
| **Q2** | | 2.55 | 1.18, 5.55 | **0.018** | 2.28 | 1.05, 4.95 | | **0.038** | | 2.26 | 1.04, 4.91 | | **0.040** | | 2.15 | 0.99, 4.68 | | 0.053 | |
| **Q3** | | 5.26 | 2.58, 10.7 | **<0.001** | 4.31 | 2.10, 8.82 | | **<0.001** | | 4.25 | 2.07, 8.73 | | **<0.001** | | 3.86 | 1.88, 7.95 | | **<0.001** | |
| **Q4** | | 9.49 | 4.76, 18.9 | **<0.001** | 6.68 | 3.29, 13.6 | | **<0.001** | | 6.70 | 3.29, 13.7 | | **<0.001** | | 5.84 | 2.86, 11.9 | | **<0.001** | |
| **P for trend** | |  |  | **<0.001** |  |  | | **<0.001** | |  |  | | **<0.001** | |  |  | | **<0.001** | |
| **Cumulative average EURO-D** | | 1.40 | 1.31, 1.49 | **<0.001** | 1.36 | 1.26, 1.47 | | **<0.001** | | 1.37 | 1.27, 1.48 | | **<0.001** | | 1.35 | 1.25, 1.47 | | **<0.001** | |
| **Quartile cumulative average EURO-D** | |  |  |  |  |  | |  | |  |  | |  | |  |  | |  | |
| **Q1** | | Reference | | | Reference | | | | | Reference | | | | | Reference | | | | |
| **Q2** | | 2.55 | 1.18, 5.55 | **0.018** | 2.28 | 1.05, 4.95 | | **0.038** | | 2.26 | 1.04, 4.91 | | **0.040** | | 2.15 | 0.99, 4.68 | | 0.053 | |
| **Q3** | | 5.26 | 2.58, 10.7 | **<0.001** | 4.31 | 2.10, 8.82 | | **<0.001** | | 4.25 | 2.07, 8.73 | | **<0.001** | | 3.86 | 1.88, 7.95 | | **<0.001** | |
| **Q4** | | 9.49 | 4.76, 18.9 | **<0.001** | 6.68 | 3.29, 13.6 | | **<0.001** | | 6.70 | 3.29, 13.7 | | **<0.001** | | 5.84 | 2.86, 11.9 | | **<0.001** | |
| **P for trend** | |  |  | **<0.001** |  |  | | **<0.001** | |  |  | | **<0.001** | |  |  | | **<0.001** | |
| **Cumulative burden** | |  |  |  |  |  | |  | |  |  | |  | |  |  | |  | |
| **<0** | | Reference | | | Reference | | | | | Reference | | | | | Reference | | | | |
| **≥0** | | 4.20 | 3.03, 5.83 | **<0.001** | 3.42 | 2.38, 4.93 | | **<0.001** | | 3.59 | 2.49, 5.18 | | **<0.001** | | 3.40 | 2.36, 4.92 | | **<0.001** | |
| **Exposure duration** | |  |  |  |  |  | |  | |  |  | |  | |  |  | |  | |
| **0 year** | | Reference | | | Reference | | | | | Reference | | | | | Reference | | | | |
| **2 years** | | 2.81 | 1.72, 4.57 | **<0.001** | 2.32 | 1.39, 3.88 | | **0.001** | | 2.41 | 1.44, 4.05 | | **<0.001** | | 2.31 | 1.38, 3.87 | | **0.001** | |
| **4 years** | | 5.30 | 3.53, 7.95 | **<0.001** | 4.25 | 2.74, 6.59 | | **<0.001** | | 4.30 | 2.76, 6.69 | | **<0.001** | | 4.03 | 2.57, 6.30 | | **<0.001** | |
| **P for trend** | |  |  | **<0.001** |  |  | | **0.0013** | |  |  | | **<0.001** | |  |  | | **<0.001** | |
| **Slope** | |  |  |  |  |  | |  | |  |  | |  | |  |  | |  | |
| **<0** | | Reference | | | Reference | | | | | Reference | | | | | Reference | | | | |
| **≥0** | | 1.34 | 0.98, 1.85 | 0.069 | 1.24 | 0.90, 1.71 | | 0.184 | | 1.24 | 0.90, 1.71 | | 0.190 | | 1.28 | 0.93, 1.77 | | 0.133 | |
| **Time course patterns** | |  |  |  |  |  | |  | |  |  | |  | |  |  | |  | |
| **Decrease-decrease** | | Reference | | | Reference | | | | | Reference | | | | | Reference | | | | |
| **Decrease-increase** | | 2.13 | 1.37, 3.31 | **<0.001** | 1.77 | 1.14, 2.75 | | **0.012** | | 1.78 | 1.14, 2.77 | | **0.011** | | 1.73 | 1.11, 2.70 | | **0.016** | |
| **Increase-decrease** | | 1.80 | 1.14, 2.83 | **0.011** | 1.38 | 0.88, 2.18 | | 0.164 | | 1.39 | 0.88, 2.19 | | 0.163 | | 1.42 | 0.90, 2.25 | | 0.131 | |
| **Increase-increase** | | 2.31 | 1.29, 4.15 | **0.005** | 1.47 | 0.81, 2.65 | | 0.206 | | 1.48 | 0.82, 2.67 | | 0.199 | | 1.36 | 0.75, 2.47 | | 0.312 | |
| CI^1^ = Confidence Interval, HR = Hazard Ratio | | | |  |  |  | |  | |  |  | |  | |  |  | |  |  |

Model 1: unadjusted

Model 2: adjusted for age, gender, marital status, education level

Model 3: adjusted for Model 2 plus smoking status, drinking status, HbA1c, TC, HDL-c, CRP

Model 4: adjusted for Model 3 plus depression, hypertension, diabetes, heart disease, stroke

Supplementary Table 8 Cox regression models for the association between the cumulative exposure and incident dementia risk in CHARLS with lagged analyses

| **Categories** | | **Model 1** | | | **Model 2** | | | | | **Model 3** | | | | | **Model 4** | | | |  |
| --- | --- | --- | --- | --- | --- | --- | --- | --- | --- | --- | --- | --- | --- | --- | --- | --- | --- | --- | --- |
|  | | **HR** | **95% CI^1^** | **p-value** | **HR** | **95% CI** | | **p-value** | | **HR** | **95% CI** | | **p-value** | | **HR** | **95% CI** | | **p-value** | |
| **Cumulative CES-D** | | 1.04 | 1.03, 1.04 | **<0.001** | 1.04 | 1.03, 1.05 | | **<0.001** | | 1.04 | 1.03, 1.05 | | **<0.001** | | 1.04 | 1.03, 1.05 | | **<0.001** | |
| **Quartile cumulative CES-D** | |  |  |  |  |  | |  | |  |  | |  | |  |  | |  | |
| **Q1** | | Reference | | | Reference | | | | | Reference | | | | | Reference | | | | |
| **Q2** | | 1.34 | 0.57, 3.19 | 0.502 | 1.22 | 0.51, 2.90 | | 0.651 | | 1.20 | 0.51, 2.86 | | 0.677 | | 1.18 | 0.50, 2.82 | | 0.706 | |
| **Q3** | | 3.89 | 1.85, 8.18 | **<0.001** | 3.55 | 1.68, 7.52 | | **<0.001** | | 3.46 | 1.64, 7.32 | | **0.001** | | 3.41 | 1.61, 7.23 | | **0.001** | |
| **Q4** | | 7.59 | 3.77, 15.3 | **<0.001** | 8.05 | 3.93, 16.5 | | **<0.001** | | 7.49 | 3.66, 15.3 | | **<0.001** | | 7.37 | 3.58, 15.2 | | **<0.001** | |
| **P for trend** | |  |  | **<0.001** |  |  | | **<0.001** | |  |  | | **<0.001** | |  |  | | **<0.001** | |
| **Cumulative average CES-D** | | 1.15 | 1.11, 1.18 | **<0.001** | 1.16 | 1.13, 1.20 | | **<0.001** | | 1.16 | 1.12, 1.20 | | **<0.001** | | 1.16 | 1.12, 1.20 | | **<0.001** | |
| **Quartile cumulative average CES-D** | |  |  |  |  |  | |  | |  |  | |  | |  |  | |  | |
| **Q1** | | Reference | | | Reference | | | | | Reference | | | | | Reference | | | | |
| **Q2** | | 1.34 | 0.57, 3.19 | 0.502 | 1.22 | 0.51, 2.90 | | 0.651 | | 1.20 | 0.51, 2.86 | | 0.677 | | 1.18 | 0.50, 2.82 | | 0.706 | |
| **Q3** | | 3.89 | 1.85, 8.18 | **<0.001** | 3.55 | 1.68, 7.52 | | **<0.001** | | 3.46 | 1.64, 7.32 | | **0.001** | | 3.41 | 1.61, 7.23 | | **0.001** | |
| **Q4** | | 7.59 | 3.77, 15.3 | **<0.001** | 8.05 | 3.93, 16.5 | | **<0.001** | | 7.49 | 3.66, 15.3 | | **<0.001** | | 7.37 | 3.58, 15.2 | | **<0.001** | |
| **P for trend** | |  |  | **<0.001** |  |  | | **<0.001** | |  |  | | **<0.001** | |  |  | | **<0.001** | |
| **Cumulative burden** | |  |  |  |  |  | |  | |  |  | |  | |  |  | |  | |
| **<0** | | Reference | | | Reference | | | | | Reference | | | | | Reference | | | | |
| **≥0** | | 4.46 | 2.78, 7.18 | **<0.001** | 4.39 | 2.71, 7.12 | | **<0.001** | | 4.22 | 2.60, 6.85 | | **<0.001** | | 4.16 | 2.56, 6.76 | | **<0.001** | |
| **Exposure duration** | |  |  |  |  |  | |  | |  |  | |  | |  |  | |  | |
| **0 year** | | Reference | | | Reference | | | | | Reference | | | | | Reference | | | | |
| **2 years** | | 2.43 | 1.44, 4.09 | **<0.001** | 2.26 | 1.34, 3.84 | | **0.002** | | 2.20 | 1.29, 3.73 | | **0.004** | | 2.19 | 1.29, 3.71 | | **0.004** | |
| **4 years** | | 3.93 | 2.58, 5.98 | **<0.001** | 4.17 | 2.70, 6.43 | | **<0.001** | | 3.90 | 2.52, 6.04 | | **<0.001** | | 3.86 | 2.49, 6.01 | | **<0.001** | |
| **P for trend** | |  |  | **<0.001** |  |  | | **<0.001** | |  |  | | **<0.001** | |  |  | | **<0.001** | |
| **Slope** | |  |  |  |  |  | |  | |  |  | |  | |  |  | |  | |
| **<0** | | Reference | | | Reference | | | | | Reference | | | | | Reference | | | | |
| **≥0** | | 1.36 | 0.94, 1.97 | 0.106 | 1.35 | 0.93, 1.96 | | 0.112 | | 1.37 | 0.94, 1.99 | | 0.099 | | 1.41 | 0.97, 2.06 | | 0.071 | |
| **Time course patterns** | |  |  |  |  |  | |  | |  |  | |  | |  |  | |  | |
| **Decrease-decrease** | | Reference | | | Reference | | | | | Reference | | | | | Reference | | | | |
| **Decrease-increase** | | 2.13 | 1.20, 3.79 | **0.010** | 2.24 | 1.26, 3.98 | | **0.006** | | 2.28 | 1.28, 4.07 | | **0.005** | | 2.27 | 1.27, 4.05 | | **0.006** | |
| **Increase-decrease** | | 2.07 | 1.16, 3.70 | **0.014** | 2.24 | 1.25, 4.00 | | **0.007** | | 2.32 | 1.29, 4.15 | | **0.005** | | 2.40 | 1.34, 4.31 | | **0.003** | |
| **Increase-increase** | | 1.68 | 0.82, 3.45 | 0.155 | 1.62 | 0.79, 3.34 | | 0.189 | | 1.66 | 0.80, 3.44 | | 0.170 | | 1.70 | 0.82, 3.52 | | 0.152 | |
| CI^1^ = Confidence Interval, HR = Hazard Ratio | | | |  |  |  | |  | |  |  | |  | |  |  | |  |  |

Model 1: unadjusted

Model 2: adjusted for age, gender, marital status, education level

Model 3: adjusted for Model 2 plus smoking status, drinking status, HbA1c, TC, HDL-c, CRP

Model 4: adjusted for Model 3 plus depression, hypertension, diabetes, heart disease, stroke

Supplementary Table 9 Logistic regression models for the association between the cumulative exposure and incident dementia risk in ELSA

| **Categories** | | **Model 1** | | | **Model 2** | | | | | **Model 3** | | | | | **Model 4** | | | |  |
| --- | --- | --- | --- | --- | --- | --- | --- | --- | --- | --- | --- | --- | --- | --- | --- | --- | --- | --- | --- |
|  | | **OR** | **95% CI^1^** | **p-value** | **OR** | **95% CI** | | **p-value** | | **OR** | **95% CI** | | **p-value** | | **OR** | **95% CI** | | **p-value** | |
| **Cumulative CES-D** | | 1.07 | 1.05, 1.09 | **<0.001** | 1.07 | 1.05, 1.10 | | **<0.001** | | 1.07 | 1.04, 1.09 | | **<0.001** | | 1.07 | 1.04, 1.09 | | **<0.001** | |
| **Quartile cumulative CES-D** | |  |  |  |  |  | |  | |  |  | |  | |  |  | |  | |
| **Q1** | | Reference | | | Reference | | | | | Reference | | | | | Reference | | | | |
| **Q2** | | 0.96 | 0.52, 1.80 | 0.909 | 0.89 | 0.48, 1.67 | | 0.713 | | 0.88 | 0.47, 1.65 | | 0.687 | | 0.86 | 0.46, 1.62 | | 0.634 | |
| **Q3** | | 1.97 | 1.14, 3.52 | **0.017** | 1.68 | 0.95, 3.03 | | 0.077 | | 1.64 | 0.93, 2.95 | | 0.094 | | 1.63 | 0.92, 2.95 | | 0.096 | |
| **Q4** | | 3.75 | 2.30, 6.38 | **<0.001** | 3.21 | 1.92, 5.57 | | **<0.001** | | 3.02 | 1.80, 5.25 | | **<0.001** | | 2.95 | 1.75, 5.15 | | **<0.001** | |
| **P for trend** | |  |  | **<0.001** |  |  | | **<0.001** | |  |  | | **<0.001** | |  |  | | **<0.001** | |
| **Cumulative average CES-D** | | 1.31 | 1.21, 1.42 | **<0.001** | 1.33 | 1.21, 1.45 | | **<0.001** | | 1.31 | 1.19, 1.44 | | **<0.001** | | 1.31 | 1.18, 1.44 | | **<0.001** | |
| **Quartile cumulative average CES-D** | |  |  |  |  |  | |  | |  |  | |  | |  |  | |  | |
| **Q1** | | Reference | | | Reference | | | | | Reference | | | | | Reference | | | | |
| **Q2** | | 0.96 | 0.52, 1.80 | 0.909 | 0.89 | 0.48, 1.67 | | 0.713 | | 0.88 | 0.47, 1.65 | | 0.687 | | 0.86 | 0.46, 1.62 | | 0.634 | |
| **Q3** | | 1.97 | 1.14, 3.52 | **0.017** | 1.68 | 0.95, 3.03 | | 0.077 | | 1.64 | 0.93, 2.95 | | 0.094 | | 1.63 | 0.92, 2.95 | | 0.096 | |
| **Q4** | | 3.75 | 2.30, 6.38 | **<0.001** | 3.21 | 1.92, 5.57 | | **<0.001** | | 3.02 | 1.80, 5.25 | | **<0.001** | | 2.95 | 1.75, 5.15 | | **<0.001** | |
| **P for trend** | |  |  | **<0.001** |  |  | | **<0.001** | |  |  | | **<0.001** | |  |  | | **<0.001** | |
| **Cumulative burden** | |  |  |  |  |  | |  | |  |  | |  | |  |  | |  | |
| **<0** | | Reference | | | Reference | | | | | Reference | | | | | Reference | | | | |
| **≥0** | | 3.04 | 2.13, 4.28 | **<0.001** | 2.78 | 1.92, 4.01 | | **<0.001** | | 2.64 | 1.81, 3.83 | | **<0.001** | | 2.62 | 1.79, 3.82 | | **<0.001** | |
| **Exposure duration** | |  |  |  |  |  | |  | |  |  | |  | |  |  | |  | |
| **0 year** | | Reference | | | Reference | | | | | Reference | | | | | Reference | | | | |
| **2 years** | | 2.05 | 1.11, 3.51 | **0.014** | 1.73 | 0.92, 3.02 | | 0.069 | | 1.63 | 0.87, 2.86 | | 0.106 | | 1.60 | 0.84, 2.82 | | 0.126 | |
| **4 years** | | 3.31 | 2.07, 5.12 | **<0.001** | 3.26 | 1.98, 5.23 | | **<0.001** | | 3.05 | 1.83, 4.91 | | **<0.001** | | 3.02 | 1.81, 4.91 | | **<0.001** | |
| **P for trend** | |  |  | **<0.001** |  |  | | **<0.001** | |  |  | | **<0.001** | |  |  | | **<0.001** | |
| **Slope** | |  |  |  |  |  | |  | |  |  | |  | |  |  | |  | |
| **<0** | | Reference | | | Reference | | | | | Reference | | | | | Reference | | | | |
| **≥0** | | 0.78 | 0.56, 1.10 | 0.155 | 0.76 | 0.54, 1.08 | | 0.127 | | 0.79 | 0.56, 1.12 | | 0.189 | | 0.78 | 0.55, 1.10 | | 0.157 | |
| **Time course patterns** | |  |  |  |  |  | |  | |  |  | |  | |  |  | |  | |
| **Decrease-decrease** | | Reference | | | Reference | | | | | Reference | | | | | Reference | | | | |
| **Decrease-increase** | | 1.67 | 1.09, 2.54 | **0.017** | 1.45 | 0.94, 2.23 | | 0.087 | | 1.43 | 0.92, 2.19 | | 0.107 | | 1.41 | 0.91, 2.17 | | 0.116 | |
| **Increase-decrease** | | 1.61 | 1.05, 2.45 | **0.026** | 1.42 | 0.92, 2.18 | | 0.105 | | 1.43 | 0.93, 2.20 | | 0.100 | | 1.44 | 0.93, 2.21 | | 0.097 | |
| **Increase-increase** | | 2.40 | 1.13, 4.59 | **0.013** | 1.70 | 0.79, 3.32 | | 0.143 | | 1.58 | 0.73, 3.10 | | 0.209 | | 1.44 | 0.66, 2.87 | | 0.326 | |
| CI^1^ = Confidence Interval, OR = Odds Ratio | | | |  |  |  | |  | |  |  | |  | |  |  | |  |  |

Model 1: unadjusted

Model 2: adjusted for age, gender, marital status, education level

Model 3: adjusted for Model 2 plus smoking status, drinking status, HbA1c, TC, HDL-c, CRP

Model 4: adjusted for Model 3 plus depression, hypertension, diabetes, heart disease, stroke

Supplementary Table 10 Logistic regression models for the association between the cumulative exposure and incident dementia risk in HRS

| **Categories** | | **Model 1** | | | **Model 2** | | | | | **Model 3** | | | | | **Model 4** | | | |  |
| --- | --- | --- | --- | --- | --- | --- | --- | --- | --- | --- | --- | --- | --- | --- | --- | --- | --- | --- | --- |
|  | | **OR** | **95% CI^1^** | **p-value** | **OR** | **95% CI** | | **p-value** | | **OR** | **95% CI** | | **p-value** | | **OR** | **95% CI** | | **p-value** | |
| **Cumulative CES-D** | | 1.08 | 1.06, 1.10 | **<0.001** | 1.10 | 1.08, 1.13 | | **<0.001** | | 1.10 | 1.08, 1.12 | | **<0.001** | | 1.09 | 1.07, 1.12 | | **<0.001** | |
| **Quartile cumulative CES-D** | |  |  |  |  |  | |  | |  |  | |  | |  |  | |  | |
| **Q1** | | Reference | | | Reference | | | | | Reference | | | | | Reference | | | | |
| **Q2** | | 4.55 | 1.69, 15.8 | **0.006** | 4.41 | 1.64, 15.3 | | **0.007** | | 4.41 | 1.63, 15.3 | | **0.008** | | 4.41 | 1.63, 15.4 | | **0.008** | |
| **Q3** | | 10.5 | 4.26, 35.1 | **<0.001** | 10.9 | 4.37, 36.5 | | **<0.001** | | 10.8 | 4.33, 36.2 | | **<0.001** | | 10.4 | 4.14, 34.8 | | **<0.001** | |
| **Q4** | | 19.6 | 8.08, 64.6 | **<0.001** | 23.4 | 9.47, 77.7 | | **<0.001** | | 22.7 | 9.16, 75.6 | | **<0.001** | | 20.2 | 8.12, 67.6 | | **<0.001** | |
| **P for trend** | |  |  | **<0.001** |  |  | | **<0.001** | |  |  | | **<0.001** | |  |  | | **<0.001** | |
| **Cumulative average CES-D** | | 1.38 | 1.28, 1.48 | **<0.001** | 1.48 | 1.36, 1.60 | | **<0.001** | | 1.47 | 1.35, 1.59 | | **<0.001** | | 1.43 | 1.31, 1.55 | | **<0.001** | |
| **Quartile cumulative average CES-D** | |  |  |  |  |  | |  | |  |  | |  | |  |  | |  | |
| **Q1** | | Reference | | | Reference | | | | | Reference | | | | | Reference | | | | |
| **Q2** | | 4.55 | 1.69, 15.8 | **0.006** | 4.41 | 1.64, 15.3 | | **0.007** | | 4.41 | 1.63, 15.3 | | **0.008** | | 4.41 | 1.63, 15.4 | | **0.008** | |
| **Q3** | | 10.5 | 4.26, 35.1 | **<0.001** | 10.9 | 4.37, 36.5 | | **<0.001** | | 10.8 | 4.33, 36.2 | | **<0.001** | | 10.4 | 4.14, 34.8 | | **<0.001** | |
| **Q4** | | 19.6 | 8.08, 64.6 | **<0.001** | 23.4 | 9.47, 77.7 | | **<0.001** | | 22.7 | 9.16, 75.6 | | **<0.001** | | 20.2 | 8.12, 67.6 | | **<0.001** | |
| **P for trend** | |  |  | **<0.001** |  |  | | **<0.001** | |  |  | | **<0.001** | |  |  | | **<0.001** | |
| **Cumulative burden** | |  |  |  |  |  | |  | |  |  | |  | |  |  | |  | |
| **<0** | | Reference | | | Reference | | | | | Reference | | | | | Reference | | | | |
| **≥0** | | 5.56 | 3.69, 8.69 | **<0.001** | 6.03 | 3.94, 9.53 | | **<0.001** | | 5.87 | 3.83, 9.30 | | **<0.001** | | 5.43 | 3.52, 8.63 | | **<0.001** | |
| **Exposure duration** | |  |  |  |  |  | |  | |  |  | |  | |  |  | |  | |
| **0 year** | | Reference | | | Reference | | | | | Reference | | | | | Reference | | | | |
| **2 years** | | 3.37 | 2.06, 5.36 | **<0.001** | 3.39 | 2.04, 5.46 | | **<0.001** | | 3.35 | 2.02, 5.41 | | **<0.001** | | 3.10 | 1.86, 5.03 | | **<0.001** | |
| **4 years** | | 3.85 | 2.59, 5.69 | **<0.001** | 4.29 | 2.83, 6.45 | | **<0.001** | | 4.12 | 2.71, 6.24 | | **<0.001** | | 3.65 | 2.38, 5.57 | | **<0.001** | |
| **P for trend** | |  |  | **<0.001** |  |  | | **<0.001** | |  |  | | **<0.001** | |  |  | | **<0.001** | |
| **Slope** | |  |  |  |  |  | |  | |  |  | |  | |  |  | |  | |
| **<0** | | Reference | | | Reference | | | | | Reference | | | | | Reference | | | | |
| **≥0** | | 0.71 | 0.50, 1.00 | 0.051 | 0.69 | 0.48, 0.98 | | **0.040** | | 0.69 | 0.48, 0.98 | | **0.037** | | 0.72 | 0.50, 1.03 | | 0.069 | |
| **Time course patterns** | |  |  |  |  |  | |  | |  |  | |  | |  |  | |  | |
| **Decrease-decrease** | | Reference | | | Reference | | | | | Reference | | | | | Reference | | | | |
| **Decrease-increase** | | 3.52 | 2.23, 5.64 | **<0.001** | 3.19 | 2.01, 5.14 | | **<0.001** | | 3.10 | 1.95, 5.01 | | **<0.001** | | 3.10 | 1.94, 5.01 | | **<0.001** | |
| **Increase-decrease** | | 2.99 | 1.86, 4.85 | **<0.001** | 2.88 | 1.78, 4.70 | | **<0.001** | | 2.82 | 1.75, 4.61 | | **<0.001** | | 2.75 | 1.70, 4.51 | | **<0.001** | |
| **Increase-increase** | | 4.65 | 2.32, 8.83 | **<0.001** | 4.03 | 1.99, 7.73 | | **<0.001** | | 3.89 | 1.92, 7.49 | | **<0.001** | | 3.63 | 1.78, 7.02 | | **<0.001** | |
| CI^1^ = Confidence Interval, OR = Odds Ratio | | | |  |  |  | |  | |  |  | |  | |  |  | |  |  |

Model 1: unadjusted

Model 2: adjusted for age, gender, marital status, education level, residence

Model 3: adjusted for Model 2 plus smoking status, drinking status, HbA1c, TC, HDL-c, CRP

Model 4: adjusted for Model 3 plus depression, hypertension, diabetes, heart disease, stroke

Supplementary Table 11 Logistic regression models for the association between the cumulative exposure and incident dementia risk in SHARE

| **Categories** | | **Model 1** | | | **Model 2** | | | | | **Model 3** | | | | | **Model 4** | | | |  |
| --- | --- | --- | --- | --- | --- | --- | --- | --- | --- | --- | --- | --- | --- | --- | --- | --- | --- | --- | --- |
|  | | **OR** | **95% CI^1^** | **p-value** | **OR** | **95% CI** | | **p-value** | | **OR** | **95% CI** | | **p-value** | | **OR** | **95% CI** | | **p-value** | |
| **Cumulative EURO-D** | | 1.10 | 1.08, 1.12 | <0.001 | 1.09 | 1.07, 1.11 | | <0.001 | | 1.09 | 1.07, 1.11 | | <0.001 | | 1.09 | 1.07, 1.11 | | <0.001 | |
| **Quartile cumulative EURO-D** | |  |  |  |  |  | |  | |  |  | |  | |  |  | |  | |
| **Q1** | | Reference | | | Reference | | | | | Reference | | | | | Reference | | | | |
| **Q2** | | 3.29 | 1.60, 7.44 | **0.002** | 2.79 | 1.35, 6.32 | | **0.008** | | 2.82 | 1.36, 6.41 | | **0.008** | | 2.72 | 1.32, 6.20 | | **0.010** | |
| **Q3** | | 7.59 | 3.94, 16.5 | **<0.001** | 6.07 | 3.12, 13.3 | | **<0.001** | | 6.15 | 3.16, 13.5 | | **<0.001** | | 5.72 | 2.93, 12.5 | | **<0.001** | |
| **Q4** | | 14.5 | 7.74, 31.0 | **<0.001** | 10.0 | 5.24, 21.8 | | **<0.001** | | 10.0 | 5.23, 21.8 | | **<0.001** | | 8.82 | 4.57, 19.2 | | **<0.001** | |
| **P for trend** | |  |  | **<0.001** |  |  | | **<0.001** | |  |  | | **<0.001** | |  |  | | **<0.001** | |
| **Cumulative average EURO-D** | | 1.46 | 1.38, 1.55 | **<0.001** | 1.43 | 1.33, 1.54 | | **<0.001** | | 1.43 | 1.33, 1.54 | | **<0.001** | | 1.40 | 1.30, 1.51 | | **<0.001** | |
| **Quartile cumulative average EURO-D** | |  |  |  |  |  | |  | |  |  | |  | |  |  | |  | |
| **Q1** | | Reference | | | Reference | | | | | Reference | | | | | Reference | | | | |
| **Q2** | | 3.29 | 1.60, 7.44 | **0.002** | 2.79 | 1.35, 6.32 | | **0.008** | | 2.82 | 1.36, 6.41 | | **0.008** | | 2.72 | 1.32, 6.20 | | **0.010** | |
| **Q3** | | 7.59 | 3.94, 16.5 | **<0.001** | 6.07 | 3.12, 13.3 | | **<0.001** | | 6.15 | 3.16, 13.5 | | **<0.001** | | 5.72 | 2.93, 12.5 | | **<0.001** | |
| **Q4** | | 14.5 | 7.74, 31.0 | **<0.001** | 10.0 | 5.24, 21.8 | | **<0.001** | | 10.0 | 5.23, 21.8 | | **<0.001** | | 8.82 | 4.57, 19.2 | | **<0.001** | |
| **P for trend** | |  |  | **<0.001** |  |  | | **<0.001** | |  |  | | **<0.001** | |  |  | | **<0.001** | |
| **Cumulative burden** | |  |  |  |  |  | |  | |  |  | |  | |  |  | |  | |
| **<0** | | Reference | | | Reference | | | | | Reference | | | | | Reference | | | | |
| **≥0** | | 4.76 | 3.50, 6.42 | **<0.001** | 3.75 | 2.66, 5.26 | | **<0.001** | | 3.73 | 2.63, 5.24 | | **<0.001** | | 3.38 | 2.38, 4.77 | | **<0.001** | |
| **Exposure duration** | |  |  |  |  |  | |  | |  |  | |  | |  |  | |  | |
| **0 year** | | Reference | | | Reference | | | | | Reference | | | | | Reference | | | | |
| **2 years** | | 2.94 | 1.82, 4.55 | **<0.001** | 2.34 | 1.41, 3.74 | | **<0.001** | | 2.34 | 1.41, 3.75 | | **<0.001** | | 2.25 | 1.35, 3.60 | | **0.001** | |
| **4 years** | | 6.21 | 4.21, 8.99 | **<0.001** | 5.12 | 3.32, 7.78 | | **<0.001** | | 5.14 | 3.31, 7.85 | | **<0.001** | | 4.47 | 2.87, 6.88 | | **<0.001** | |
| **P for trend** | |  |  | **<0.001** |  |  | | **<0.001** | |  |  | | **<0.001** | |  |  | | **<0.001** | |
| **Slope** | |  |  |  |  |  | |  | |  |  | |  | |  |  | |  | |
| **<0** | | Reference | | | Reference | | | | | Reference | | | | | Reference | | | | |
| **≥0** | | 1.28 | 0.96, 1.71 | 0.094 | 1.07 | 0.80, 1.44 | | 0.641 | | 1.08 | 0.81, 1.46 | | 0.598 | | 1.12 | 0.83, 1.51 | | 0.472 | |
| **Time course patterns** | |  |  |  |  |  | |  | |  |  | |  | |  |  | |  | |
| **Decrease-decrease** | | Reference | | | Reference | | | | | Reference | | | | | Reference | | | | |
| **Decrease-increase** | | 2.38 | 1.61, 3.58 | **<0.001** | 1.93 | 1.29, 2.93 | | **0.002** | | 1.91 | 1.28, 2.91 | | **0.002** | | 1.85 | 1.23, 2.82 | | **0.003** | |
| **Increase-decrease** | | 2.10 | 1.40, 3.18 | **<0.001** | 1.66 | 1.09, 2.53 | | **0.018** | | 1.62 | 1.07, 2.48 | | **0.025** | | 1.61 | 1.06, 2.47 | | **0.028** | |
| **Increase-increase** | | 2.86 | 1.68, 4.78 | **<0.001** | 1.80 | 1.04, 3.06 | | **0.032** | | 1.83 | 1.06, 3.11 | | **0.028** | | 1.76 | 1.01, 3.01 | | **0.042** | |
| CI^1^ = Confidence Interval, OR = Odds Ratio | | | |  |  |  | |  | |  |  | |  | |  |  | |  |  |

Model 1: unadjusted

Model 2: adjusted for age, gender, marital status, education level, residence

Model 3: adjusted for Model 2 plus smoking status, drinking status, HbA1c, TC, HDL-c, CRP

Model 4: adjusted for Model 3 plus depression, hypertension, diabetes, heart disease, stroke

Supplementary Table 12 Logistic regression models for the association between the cumulative exposure and incident dementia risk in CHARLS

| **Categories** | | **Model 1** | | | **Model 2** | | | | | **Model 3** | | | | | **Model 4** | | | |  |
| --- | --- | --- | --- | --- | --- | --- | --- | --- | --- | --- | --- | --- | --- | --- | --- | --- | --- | --- | --- |
|  | | **OR** | **95% CI^1^** | **p-value** | **OR** | **95% CI** | | **p-value** | | **OR** | **95% CI** | | **p-value** | | **OR** | **95% CI** | | **p-value** | |
| **Cumulative CES-D** | | 1.03 | 1.03, 1.04 | **<0.001** | 1.04 | 1.03, 1.04 | | **<0.001** | | 1.04 | 1.03, 1.04 | | **<0.001** | | 1.04 | 1.03, 1.04 | | **<0.001** | |
| **Quartile cumulative CES-D** | |  |  |  |  |  | |  | |  |  | |  | |  |  | |  | |
| **Q1** | | Reference | | | Reference | | | | | Reference | | | | | Reference | | | | |
| **Q2** | | 1.73 | 0.97, 3.15 | 0.065 | 1.63 | 0.91, 2.98 | | 0.105 | | 1.64 | 0.91, 3.00 | | 0.102 | | 1.57 | 0.87, 2.88 | | 0.138 | |
| **Q3** | | 2.76 | 1.64, 4.82 | **<0.001** | 2.71 | 1.60, 4.79 | | **<0.001** | | 2.72 | 1.60, 4.80 | | **<0.001** | | 2.60 | 1.53, 4.61 | | **<0.001** | |
| **Q4** | | 7.22 | 4.52, 12.2 | **<0.001** | 7.96 | 4.89, 13.6 | | **<0.001** | | 7.75 | 4.76, 13.3 | | **<0.001** | | 7.19 | 4.38, 12.4 | | **<0.001** | |
| **P for trend** | |  |  | **<0.001** |  |  | | **<0.001** | |  |  | | **<0.001** | |  |  | | **<0.001** | |
| **Cumulative average CES-D** | | 1.14 | 1.12, 1.17 | **<0.001** | 1.16 | 1.13, 1.19 | | **<0.001** | | 1.16 | 1.13, 1.19 | | **<0.001** | | 1.15 | 1.12, 1.18 | | **<0.001** | |
| **Quartile cumulative average CES-D** | |  |  |  |  |  | |  | |  |  | |  | |  |  | |  | |
| **Q1** | | Reference | | | Reference | | | | | Reference | | | | | Reference | | | | |
| **Q2** | | 1.73 | 0.97, 3.15 | 0.065 | 1.63 | 0.91, 2.98 | | 0.105 | | 1.64 | 0.91, 3.00 | | 0.102 | | 1.57 | 0.87, 2.88 | | 0.138 | |
| **Q3** | | 2.76 | 1.64, 4.82 | **<0.001** | 2.71 | 1.60, 4.79 | | **<0.001** | | 2.72 | 1.60, 4.80 | | **<0.001** | | 2.60 | 1.53, 4.61 | | **<0.001** | |
| **Q4** | | 7.22 | 4.52, 12.2 | **<0.001** | 7.96 | 4.89, 13.6 | | **<0.001** | | 7.75 | 4.76, 13.3 | | **<0.001** | | 7.19 | 4.38, 12.4 | | **<0.001** | |
| **P for trend** | |  |  | **<0.001** |  |  | | **<0.001** | |  |  | | **<0.001** | |  |  | | **<0.001** | |
| **Cumulative burden** | |  |  |  |  |  | |  | |  |  | |  | |  |  | |  | |
| **<0** | | Reference | | | Reference | | | | | Reference | | | | | Reference | | | | |
| **≥0** | | 3.59 | 2.59, 5.06 | **<0.001** | 3.71 | 2.65, 5.29 | | **<0.001** | | 3.66 | 2.61, 5.23 | | **<0.001** | | 3.49 | 2.48, 5.00 | | **<0.001** | |
| **Exposure duration** | |  |  |  |  |  | |  | |  |  | |  | |  |  | |  | |
| **0 year** | | Reference | | | Reference | | | | | Reference | | | | | Reference | | | | |
| **2 years** | | 1.88 | 1.24, 2.79 | **0.002** | 1.88 | 1.23, 2.83 | | **0.003** | | 1.85 | 1.21, 2.79 | | **0.004** | | 1.81 | 1.18, 2.73 | | **0.005** | |
| **4 years** | | 3.90 | 2.87, 5.34 | **<0.001** | 4.33 | 3.14, 6.02 | | **<0.001** | | 4.20 | 3.04, 5.85 | | **<0.001** | | 3.99 | 2.87, 5.58 | | **<0.001** | |
| **P for trend** | |  |  | **<0.001** |  |  | | **<0.001** | |  |  | | **<0.001** | |  |  | | **<0.001** | |
| **Slope** | |  |  |  |  |  | |  | |  |  | |  | |  |  | |  | |
| **<0** | | Reference | | | Reference | | | | | Reference | | | | | Reference | | | | |
| **≥0** | | 1.28 | 0.97, 1.69 | 0.077 | 1.35 | 1.02, 1.78 | | **0.038** | | 1.36 | 1.02, 1.80 | | **0.033** | | 1.39 | 1.04, 1.84 | | **0.024** | |
| **Time course patterns** | |  |  |  |  |  | |  | |  |  | |  | |  |  | |  | |
| **Decrease-decrease** | | Reference | | | Reference | | | | | Reference | | | | | Reference | | | | |
| **Decrease-increase** | | 2.11 | 1.40, 3.26 | **<0.001** | 2.22 | 1.47, 3.45 | | **<0.001** | | 2.25 | 1.49, 3.50 | | **<0.001** | | 2.21 | 1.45, 3.44 | | **<0.001** | |
| **Increase-decrease** | | 1.77 | 1.16, 2.76 | **0.010** | 1.91 | 1.25, 3.00 | | **0.004** | | 1.98 | 1.29, 3.11 | | **0.002** | | 2.02 | 1.31, 3.18 | | **0.002** | |
| **Increase-increase** | | 1.91 | 1.15, 3.18 | **0.012** | 1.99 | 1.19, 3.34 | | **0.009** | | 2.00 | 1.19, 3.37 | | **0.008** | | 2.05 | 1.21, 3.45 | | **0.007** | |
| CI^1^ = Confidence Interval, OR = Odds Ratio | | | |  |  |  | |  | |  |  | |  | |  |  | |  |  |

Model 1: unadjusted

Model 2: adjusted for age, gender, marital status, education level, residence

Model 3: adjusted for Model 2 plus smoking status, drinking status, HbA1c, TC, HDL-c, CRP

Model 4: adjusted for Model 3 plus depression, hypertension, diabetes, heart disease, stroke

Supplementary Table 13 Cox regression models for the association between the z-standardized cumulative exposure and incident dementia risk in ELSA

| **Categories** | | **Model 1** | | | **Model 2** | | | | | **Model 3** | | | | | **Model 4** | | | |  |
| --- | --- | --- | --- | --- | --- | --- | --- | --- | --- | --- | --- | --- | --- | --- | --- | --- | --- | --- | --- |
|  | | **HR** | **95% CI^1^** | **p-value** | **HR** | **95% CI** | | **p-value** | | **HR** | **95% CI** | | **p-value** | | **HR** | **95% CI** | | **p-value** | |
| **Cumulative CES-D** | | 1.13 | 1.09, 1.16 | **<0.001** | 1.12 | 1.08, 1.16 | | **<0.001** | | 1.11 | 1.07, 1.16 | | **<0.001** | | 1.11 | 1.07, 1.16 | | **<0.001** | |
| **Quartile cumulative CES-D** | |  |  |  |  |  | |  | |  |  | |  | |  |  | |  | |
| **Q1** | | Reference | | | Reference | | | | | Reference | | | | | Reference | | | | |
| **Q2** | | 1.08 | 0.57, 2.02 | 0.819 | 1.01 | 0.54, 1.89 | | 0.979 | | 0.99 | 0.52, 1.86 | | 0.969 | | 0.96 | 0.51, 1.81 | | 0.899 | |
| **Q3** | | 1.84 | 1.06, 3.19 | **0.030** | 1.52 | 0.86, 2.67 | | 0.147 | | 1.48 | 0.84, 2.61 | | 0.170 | | 1.49 | 0.85, 2.64 | | 0.167 | |
| **Q4** | | 3.56 | 2.16, 5.87 | **<0.001** | 2.84 | 1.65, 4.87 | | **<0.001** | | 2.63 | 1.53, 4.53 | | **<0.001** | | 2.58 | 1.50, 4.46 | | **<0.001** | |
| **P for trend** | |  |  | **<0.001** |  |  | | **<0.001** | |  |  | | **<0.001** | |  |  | | **<0.001** | |
| **Cumulative average CES-D** | | 1.61 | 1.42, 1.83 | **<0.001** | 1.57 | 1.34, 1.84 | | **<0.001** | | 1.52 | 1.30, 1.79 | | **<0.001** | | 1.52 | 1.30, 1.79 | | **<0.001** | |
| **Quartile cumulative average CES-D** | |  |  |  |  |  | |  | |  |  | |  | |  |  | |  | |
| **Q1** | | Reference | | | Reference | | | | | Reference | | | | | Reference | | | | |
| **Q2** | | 1.08 | 0.57, 2.02 | 0.819 | 1.01 | 0.54, 1.89 | | 0.979 | | 0.99 | 0.52, 1.86 | | 0.969 | | 0.96 | 0.51, 1.81 | | 0.899 | |
| **Q3** | | 1.84 | 1.06, 3.19 | **0.030** | 1.52 | 0.86, 2.67 | | 0.147 | | 1.48 | 0.84, 2.61 | | 0.170 | | 1.49 | 0.85, 2.64 | | 0.167 | |
| **Q4** | | 3.56 | 2.16, 5.87 | **<0.001** | 2.84 | 1.65, 4.87 | | **<0.001** | | 2.63 | 1.53, 4.53 | | **<0.001** | | 2.58 | 1.50, 4.46 | | **<0.001** | |
| **P for trend** | |  |  | **<0.001** |  |  | | **<0.001** | |  |  | | **<0.001** | |  |  | | **<0.001** | |
| **Cumulative burden** | |  |  |  |  |  | |  | |  |  | |  | |  |  | |  | |
| **<0** | | Reference | | | Reference | | | | | Reference | | | | | Reference | | | | |
| **≥0** | | 2.84 | 2.04, 3.96 | **<0.001** | 2.45 | 1.72, 3.50 | | **<0.001** | | 2.31 | 1.61, 3.31 | | **<0.001** | | 2.33 | 1.61, 3.35 | | **<0.001** | |
| **Exposure duration** | |  |  |  |  |  | |  | |  |  | |  | |  |  | |  | |
| **0 year** | | Reference | | | Reference | | | | | Reference | | | | | Reference | | | | |
| **2 years** | | 1.86 | 1.14, 3.02 | **0.012** | 1.55 | 0.95, 2.51 | | 0.078 | | 1.43 | 0.88, 2.34 | | 0.147 | | 1.40 | 0.85, 2.31 | | 0.185 | |
| **4 years** | | 3.75 | 2.55, 5.51 | **<0.001** | 3.30 | 2.18, 5.00 | | **<0.001** | | 3.08 | 2.03, 4.68 | | **<0.001** | | 3.08 | 2.02, 4.70 | | **<0.001** | |
| **P for trend** | |  |  | **<0.001** |  |  | | **<0.001** | |  |  | | **<0.001** | |  |  | | **<0.001** | |
| **Slope** | |  |  |  |  |  | |  | |  |  | |  | |  |  | |  | |
| **<0** | | Reference | | | Reference | | | | | Reference | | | | | Reference | | | | |
| **≥0** | | 1.56 | 1.12, 2.18 | **0.009** | 1.22 | 0.87, 1.72 | | 0.255 | | 1.23 | 0.88, 1.73 | | 0.229 | | 1.20 | 0.85, 1.70 | | 0.301 | |
| **Time course patterns** | |  |  |  |  |  | |  | |  |  | |  | |  |  | |  | |
| **Decrease-decrease** | | Reference | | | Reference | | | | | Reference | | | | | Reference | | | | |
| **Decrease-increase** | | 1.67 | 1.08, 2.56 | **0.020** | 1.38 | 0.89, 2.15 | | 0.153 | | 1.37 | 0.88, 2.14 | | 0.161 | | 1.40 | 0.90, 2.18 | | 0.136 | |
| **Increase-decrease** | | 1.70 | 1.12, 2.56 | **0.012** | 1.42 | 0.93, 2.16 | | 0.105 | | 1.40 | 0.92, 2.13 | | 0.117 | | 1.41 | 0.92, 2.16 | | 0.113 | |
| **Increase-increase** | | 2.55 | 1.36, 4.78 | **0.004** | 1.69 | 0.88, 3.24 | | 0.112 | | 1.59 | 0.83, 3.04 | | 0.160 | | 1.46 | 0.74, 2.89 | | 0.270 | |
| CI^1^ = Confidence Interval, HR = Hazard Ratio | | | |  |  |  | |  | |  |  | |  | |  |  | |  |  |

Model 1: unadjusted

Model 2: adjusted for age, gender, marital status, education level

Model 3: adjusted for Model 2 plus smoking status, drinking status, HbA1c, TC, HDL-c, CRP

Model 4: adjusted for Model 3 plus depression, hypertension, diabetes, heart disease, stroke

Supplementary Table 14 Cox regression models for the association between the z-standardized cumulative exposure and incident dementia risk in HRS

| **Categories** | | **Model 1** | | | **Model 2** | | | | | **Model 3** | | | | | **Model 4** | | | |  |
| --- | --- | --- | --- | --- | --- | --- | --- | --- | --- | --- | --- | --- | --- | --- | --- | --- | --- | --- | --- |
|  | | **HR** | **95% CI^1^** | **p-value** | **HR** | **95% CI** | | **p-value** | | **HR** | **95% CI** | | **p-value** | | **HR** | **95% CI** | | **p-value** | |
| **Cumulative CES-D** | | 1.17 | 1.13, 1.20 | **<0.001** | 1.20 | 1.15, 1.24 | | **<0.001** | | 1.19 | 1.15, 1.23 | | **<0.001** | | 1.18 | 1.14, 1.22 | | **<0.001** | |
| **Quartile cumulative CES-D** | |  |  |  |  |  | |  | |  |  | |  | |  |  | |  | |
| **Q1** | | Reference | | | Reference | | | | | Reference | | | | | Reference | | | | |
| **Q2** | | 4.29 | 1.44, 12.7 | **0.009** | 3.93 | 1.32, 11.7 | | **0.014** | | 4.00 | 1.34, 11.9 | | **0.013** | | 3.91 | 1.30, 11.7 | | **0.015** | |
| **Q3** | | 9.21 | 3.28, 25.9 | **<0.001** | 9.18 | 3.24, 26.0 | | **<0.001** | | 8.99 | 3.15, 25.7 | | **<0.001** | | 8.64 | 2.99, 24.9 | | **<0.001** | |
| **Q4** | | 19.1 | 6.98, 52.2 | **<0.001** | 20.6 | 7.37, 57.7 | | **<0.001** | | 20.5 | 7.30, 57.3 | | **<0.001** | | 18.1 | 6.40, 51.2 | | **<0.001** | |
| **P for trend** | |  |  | **<0.001** |  |  | | **<0.001** | |  |  | | **<0.001** | |  |  | | **<0.001** | |
| **Cumulative average CES-D** | | 1.85 | 1.64, 2.08 | **<0.001** | 2.04 | 1.77, 2.36 | | **<0.001** | | 2.02 | 1.76, 2.33 | | **<0.001** | | 1.92 | 1.66, 2.23 | | **<0.001** | |
| **Quartile cumulative average CES-D** | |  |  |  |  |  | |  | |  |  | |  | |  |  | |  | |
| **Q1** | | Reference | | | Reference | | | | | Reference | | | | | Reference | | | | |
| **Q2** | | 4.29 | 1.44, 12.7 | **0.009** | 3.93 | 1.32, 11.7 | | **0.014** | | 4.00 | 1.34, 11.9 | | **0.013** | | 3.91 | 1.30, 11.7 | | **0.015** | |
| **Q3** | | 9.21 | 3.28, 25.9 | **<0.001** | 9.18 | 3.24, 26.0 | | **<0.001** | | 8.99 | 3.15, 25.7 | | **<0.001** | | 8.64 | 2.99, 24.9 | | **<0.001** | |
| **Q4** | | 19.1 | 6.98, 52.2 | **<0.001** | 20.6 | 7.37, 57.7 | | **<0.001** | | 20.5 | 7.30, 57.3 | | **<0.001** | | 18.1 | 6.40, 51.2 | | **<0.001** | |
| **P for trend** | |  |  | **<0.001** |  |  | | **<0.001** | |  |  | | **<0.001** | |  |  | | **<0.001** | |
| **Cumulative burden** | |  |  |  |  |  | |  | |  |  | |  | |  |  | |  | |
| **<0** | | Reference | | | Reference | | | | | Reference | | | | | Reference | | | | |
| **≥0** | | 3.01 | 2.05, 4.44 | **<0.001** | 3.72 | 2.46, 5.60 | | **<0.001** | | 3.61 | 2.40, 5.43 | | **<0.001** | | 3.18 | 2.09, 4.83 | | **<0.001** | |
| **Exposure duration** | |  |  |  |  |  | |  | |  |  | |  | |  |  | |  | |
| **0 year** | | Reference | | | Reference | | | | | Reference | | | | | Reference | | | | |
| **2 years** | | 1.89 | 0.99, 3.62 | 0.053 | 2.06 | 1.06, 4.00 | | **0.032** | | 2.01 | 1.03, 3.90 | | **0.040** | | 1.88 | 0.94, 3.75 | | 0.074 | |
| **4 years** | | 3.48 | 2.13, 5.66 | **<0.001** | 4.16 | 2.44, 7.10 | | **<0.001** | | 3.97 | 2.36, 6.68 | | **<0.001** | | 3.44 | 2.03, 5.83 | | **<0.001** | |
| **P for trend** | |  |  | **<0.001** |  |  | | **<0.001** | |  |  | | **<0.001** | |  |  | | **<0.001** | |
| **Slope** | |  |  |  |  |  | |  | |  |  | |  | |  |  | |  | |
| **<0** | | Reference | | | Reference | | | | | Reference | | | | | Reference | | | | |
| **≥0** | | 2.15 | 1.53, 3.04 | **<0.001** | 1.90 | 1.34, 2.69 | | **<0.001** | | 1.80 | 1.26, 2.58 | | **0.001** | | 1.78 | 1.24, 2.55 | | **0.002** | |
| **Time course patterns** | |  |  |  |  |  | |  | |  |  | |  | |  |  | |  | |
| **Decrease-decrease** | | Reference | | | Reference | | | | | Reference | | | | | Reference | | | | |
| **Decrease-increase** | | 3.50 | 2.21, 5.52 | **<0.001** | 3.04 | 1.91, 4.82 | | **<0.001** | | 2.95 | 1.84, 4.71 | | **<0.001** | | 2.94 | 1.84, 4.69 | | **<0.001** | |
| **Increase-decrease** | | 2.85 | 1.78, 4.57 | **<0.001** | 2.67 | 1.66, 4.29 | | **<0.001** | | 2.59 | 1.60, 4.20 | | **<0.001** | | 2.50 | 1.55, 4.04 | | **<0.001** | |
| **Increase-increase** | | 4.67 | 2.46, 8.88 | **<0.001** | 2.67 | 1.66, 4.29 | | **<0.001** | | 3.61 | 1.86, 7.01 | | **<0.001** | | 3.27 | 1.70, 6.28 | | **<0.001** | |
| CI^1^ = Confidence Interval, HR = Hazard Ratio | | | |  |  |  | |  | |  |  | |  | |  |  | |  |  |

Model 1: unadjusted

Model 2: adjusted for age, gender, marital status, education level

Model 3: adjusted for Model 2 plus smoking status, drinking status, HbA1c, TC, HDL-c, CRP

Model 4: adjusted for Model 3 plus depression, hypertension, diabetes, heart disease, stroke

Supplementary Table 15 Cox regression models for the association between the z-standardized cumulative exposure and incident dementia risk in SHARE

| **Categories** | | **Model 1** | | | **Model 2** | | | | | **Model 3** | | | | | **Model 4** | | | |  |
| --- | --- | --- | --- | --- | --- | --- | --- | --- | --- | --- | --- | --- | --- | --- | --- | --- | --- | --- | --- |
|  | | **HR** | **95% CI^1^** | **p-value** | **HR** | **95% CI** | | **p-value** | | **HR** | **95% CI** | | **p-value** | | **HR** | **95% CI** | | **p-value** | |
| **Cumulative EURO-D** | | 1.21 | 1.17, 1.24 | **<0.001** | 1.18 | 1.14, 1.23 | | **<0.001** | | 1.18 | 1.14, 1.23 | | **<0.001** | | 1.17 | 1.13, 1.21 | | **<0.001** | |
| **Quartile cumulative EURO-D** | |  |  |  |  |  | |  | |  |  | |  | |  |  | |  | |
| **Q1** | | Reference | | | Reference | | | | | Reference | | | | | Reference | | | | |
| **Q2** | | 3.13 | 1.41, 6.95 | **0.005** | 2.82 | 1.26, 6.28 | | **0.011** | | 2.84 | 1.28, 6.33 | | **0.011** | | 2.67 | 1.20, 5.95 | | **0.016** | |
| **Q3** | | 6.50 | 3.10, 13.6 | **<0.001** | 5.44 | 2.57, 11.5 | | **<0.001** | | 5.42 | 2.56, 11.5 | | **<0.001** | | 4.96 | 2.31, 10.7 | | **<0.001** | |
| **Q4** | | 12.6 | 6.17, 25.8 | **<0.001** | 9.01 | 4.27, 19.0 | | **<0.001** | | 8.78 | 4.14, 18.6 | | **<0.001** | | 7.57 | 3.54, 16.2 | | **<0.001** | |
| **P for trend** | |  |  | **<0.001** |  |  | | **<0.001** | |  |  | | **<0.001** | |  |  | | **<0.001** | |
| **Cumulative average EURO-D** | | 2.11 | 1.90, 2.36 | **<0.001** | 1.96 | 1.70, 2.26 | | **<0.001** | | 1.96 | 1.69, 2.26 | | **<0.001** | | 1.86 | 1.61, 2.14 | | **<0.001** | |
| **Quartile cumulative average EURO-D** | |  |  |  |  |  | |  | |  |  | |  | |  |  | |  | |
| **Q1** | | Reference | | | Reference | | | | | Reference | | | | | Reference | | | | |
| **Q2** | | 3.13 | 1.41, 6.95 | **0.005** | 2.82 | 1.26, 6.28 | | **0.011** | | 2.84 | 1.28, 6.33 | | **0.011** | | 2.67 | 1.20, 5.95 | | **0.016** | |
| **Q3** | | 6.50 | 3.10, 13.6 | **<0.001** | 5.44 | 2.57, 11.5 | | **<0.001** | | 5.42 | 2.56, 11.5 | | **<0.001** | | 4.96 | 2.31, 10.7 | | **<0.001** | |
| **Q4** | | 12.6 | 6.17, 25.8 | **<0.001** | 9.01 | 4.27, 19.0 | | **<0.001** | | 8.78 | 4.14, 18.6 | | **<0.001** | | 7.57 | 3.54, 16.2 | | **<0.001** | |
| **P for trend** | |  |  | **<0.001** |  |  | | **<0.001** | |  |  | | **<0.001** | |  |  | | **<0.001** | |
| **Cumulative burden** | |  |  |  |  |  | |  | |  |  | |  | |  |  | |  | |
| **<0** | | Reference | | | Reference | | | | | Reference | | | | | Reference | | | | |
| **≥0** | | 4.35 | 3.26, 5.80 | **<0.001** | 3.23 | 2.33, 4.46 | | **<0.001** | | 3.15 | 2.26, 4.38 | | **<0.001** | | 2.87 | 2.07, 3.97 | | **<0.001** | |
| **Exposure duration** | |  |  |  |  |  | |  | |  |  | |  | |  |  | |  | |
| **0 year** | | Reference | | | Reference | | | | | Reference | | | | | Reference | | | | |
| **2 years** | | 2.69 | 1.74, 4.14 | **<0.001** | 2.16 | 1.34, 3.47 | | **0.001** | | 2.10 | 1.31, 3.36 | | **0.002** | | 1.95 | 1.22, 3.11 | | **0.005** | |
| **4 years** | | 5.34 | 3.77, 7.57 | **<0.001** | 4.12 | 2.80, 6.08 | | **<0.001** | | 4.12 | 2.78, 6.12 | | **<0.001** | | 3.67 | 2.53, 5.34 | | **<0.001** | |
| **P for trend** | |  |  | **<0.001** |  |  | | **<0.001** | |  |  | | **<0.001** | |  |  | | **<0.001** | |
| **Slope** | |  |  |  |  |  | |  | |  |  | |  | |  |  | |  | |
| **<0** | | Reference | | | Reference | | | | | Reference | | | | | Reference | | | | |
| **≥0** | | 1.67 | 1.26, 2.21 | **<0.001** | 1.36 | 1.02, 1.80 | | **0.033** | | 1.37 | 1.03, 1.81 | | **0.029** | | 1.38 | 1.04, 1.83 | | **0.028** | |
| **Time course patterns** | |  |  |  |  |  | |  | |  |  | |  | |  |  | |  | |
| **Decrease-decrease** | | Reference | | | Reference | | | | | Reference | | | | | Reference | | | | |
| **Decrease-increase** | | 1.90 | 1.28, 2.81 | **0.001** | 1.63 | 1.11, 2.41 | | **0.013** | | 1.62 | 1.09, 2.40 | | **0.017** | | 1.58 | 1.06, 2.35 | | **0.026** | |
| **Increase-decrease** | | 1.14 | 0.76, 1.70 | 0.520 | 1.02 | 0.68, 1.51 | | 0.935 | | 1.00 | 0.67, 1.50 | | 0.992 | | 1.02 | 0.68, 1.54 | | 0.913 | |
| **Increase-increase** | | 1.32 | 0.81, 2.17 | 0.270 | 1.00 | 0.61, 1.66 | | 0.990 | | 1.02 | 0.62, 1.68 | | 0.936 | | 0.97 | 0.58, 1.62 | | 0.895 | |
| CI^1^ = Confidence Interval, HR = Hazard Ratio | | | |  |  |  | |  | |  |  | |  | |  |  | |  |  |

Model 1: unadjusted

Model 2: adjusted for age, gender, marital status, education level, residence

Model 3: adjusted for Model 2 plus smoking status, drinking status, HbA1c, TC, HDL-c, CRP

Model 4: adjusted for Model 3 plus depression, hypertension, diabetes, heart disease, stroke

Supplementary Table 16 Cox regression models for the association between the z-standardized cumulative exposure and incident dementia risk in CHARLS

| **Categories** | | **Model 1** | | | **Model 2** | | | | | **Model 3** | | | | | **Model 4** | | | |  |
| --- | --- | --- | --- | --- | --- | --- | --- | --- | --- | --- | --- | --- | --- | --- | --- | --- | --- | --- | --- |
|  | | **HR** | **95% CI^1^** | **p-value** | **HR** | **95% CI** | | **p-value** | | **HR** | **95% CI** | | **p-value** | | **HR** | **95% CI** | | **p-value** | |
| **Cumulative CES-D** | | 1.20 | 1.17, 1.24 | **<0.001** | 1.22 | 1.18, 1.26 | | **<0.001** | | 1.22 | 1.18, 1.25 | | **<0.001** | | 1.21 | 1.17, 1.24 | | **<0.001** | |
| **Quartile cumulative CES-D** | |  |  |  |  |  | |  | |  |  | |  | |  |  | |  | |
| **Q1** | | Reference | | | Reference | | | | | Reference | | | | | Reference | | | | |
| **Q2** | | 1.57 | 0.89, 2.80 | 0.122 | 1.48 | 0.84, 2.62 | | 0.177 | | 1.46 | 0.82, 2.60 | | 0.195 | | 1.37 | 0.77, 2.45 | | 0.286 | |
| **Q3** | | 2.79 | 1.64, 4.72 | **<0.001** | 2.64 | 1.56, 4.46 | | **<0.001** | | 2.61 | 1.55, 4.40 | | **<0.001** | | 2.46 | 1.46, 4.17 | | **<0.001** | |
| **Q4** | | 6.62 | 4.09, 10.7 | **<0.001** | 6.90 | 4.27, 11.1 | | **<0.001** | | 6.65 | 4.11, 10.8 | | **<0.001** | | 6.12 | 3.76, 9.96 | | **<0.001** | |
| **P for trend** | |  |  | **<0.001** |  |  | | **<0.001** | |  |  | | **<0.001** | |  |  | | **<0.001** | |
| **Cumulative average CES-D** | | 2.10 | 1.88, 2.35 | **<0.001** | 2.21 | 1.96, 2.49 | | **<0.001** | | 2.19 | 1.94, 2.47 | | **<0.001** | | 2.12 | 1.88, 2.39 | | **<0.001** | |
| **Quartile cumulative average CES-D** | |  |  |  |  |  | |  | |  |  | |  | |  |  | |  | |
| **Q1** | | Reference | | | Reference | | | | | Reference | | | | | Reference | | | | |
| **Q2** | | 1.57 | 0.89, 2.80 | 0.122 | 1.48 | 0.84, 2.62 | | 0.177 | | 1.46 | 0.82, 2.60 | | 0.195 | | 1.37 | 0.77, 2.45 | | 0.286 | |
| **Q3** | | 2.79 | 1.64, 4.72 | **<0.001** | 2.64 | 1.56, 4.46 | | **<0.001** | | 2.61 | 1.55, 4.40 | | **<0.001** | | 2.46 | 1.46, 4.17 | | **<0.001** | |
| **Q4** | | 6.62 | 4.09, 10.7 | **<0.001** | 6.90 | 4.27, 11.1 | | **<0.001** | | 6.65 | 4.11, 10.8 | | **<0.001** | | 6.12 | 3.76, 9.96 | | **<0.001** | |
| **P for trend** | |  |  | **<0.001** |  |  | | **<0.001** | |  |  | | **<0.001** | |  |  | | **<0.001** | |
| **Cumulative burden** | |  |  |  |  |  | |  | |  |  | |  | |  |  | |  | |
| **<0** | | Reference | | | Reference | | | | | Reference | | | | | Reference | | | | |
| **≥0** | | 3.57 | 2.64, 4.84 | **<0.001** | 3.55 | 2.63, 4.79 | | **<0.001** | | 3.50 | 2.59, 4.73 | | **<0.001** | | 3.34 | 2.46, 4.53 | | **<0.001** | |
| **Exposure duration** | |  |  |  |  |  | |  | |  |  | |  | |  |  | |  | |
| **0 year** | | Reference | | | Reference | | | | | Reference | | | | | Reference | | | | |
| **2 years** | | 2.00 | 1.36, 2.93 | **<0.001** | 2.04 | 1.39, 2.99 | | **<0.001** | | 2.01 | 1.36, 2.96 | | **<0.001** | | 1.94 | 1.31, 2.87 | | **<0.001** | |
| **4 years** | | 4.19 | 3.12, 5.64 | **<0.001** | 4.27 | 3.16, 5.78 | | **<0.001** | | 4.14 | 3.05, 5.61 | | **<0.001** | | 3.94 | 2.90, 5.37 | | **<0.001** | |
| **P for trend** | |  |  | **<0.001** |  |  | | **<0.001** | |  |  | | **<0.001** | |  |  | | **<0.001** | |
| **Slope** | |  |  |  |  |  | |  | |  |  | |  | |  |  | |  | |
| **<0** | | Reference | | | Reference | | | | | Reference | | | | | Reference | | | | |
| **≥0** | | 1.04 | 0.80, 1.36 | 0.761 | 1.06 | 0.81, 1.38 | | 0.684 | | 1.07 | 0.82, 1.40 | | 0.616 | | 1.12 | 0.86, 1.47 | | 0.404 | |
| **Time course patterns** | |  |  |  |  |  | |  | |  |  | |  | |  |  | |  | |
| **Decrease-decrease** | | Reference | | | Reference | | | | | Reference | | | | | Reference | | | | |
| **Decrease-increase** | | 1.53 | 0.98, 2.39 | 0.062 | 1.57 | 1.00, 2.45 | | **0.048** | | 1.58 | 1.01, 2.47 | | **0.045** | | 1.57 | 1.00, 2.46 | | 0.051 | |
| **Increase-decrease** | | 1.42 | 0.90, 2.23 | 0.130 | 1.49 | 0.95, 2.35 | | 0.085 | | 1.50 | 0.95, 2.37 | | 0.079 | | 1.54 | 0.97, 2.43 | | 0.066 | |
| **Increase-increase** | | 1.48 | 0.89, 2.44 | 0.128 | 1.47 | 0.88, 2.43 | | 0.138 | | 1.46 | 0.88, 2.43 | | 0.145 | | 1.52 | 0.91, 2.53 | | 0.110 | |
| CI^1^ = Confidence Interval, HR = Hazard Ratio | | | |  |  |  | |  | |  |  | |  | |  |  | |  |  |

Model 1: unadjusted

Model 2: adjusted for age, gender, marital status, education level

Model 3: adjusted for Model 2 plus smoking status, drinking status, HbA1c, TC, HDL-c, CRP

Model 4: adjusted for Model 3 plus depression, hypertension, diabetes, heart disease, stroke

Formula of the indices definition:

$$\mathrm{CumDS}=\left( {\mathrm{CES}-D/EURO-D}_{first}+{\mathrm{CES}-D/EURO-D}_{second} \right)/2\times{time}_{second-first}+ \left( {\mathrm{CES}-D/EURO-D}_{second}+{\mathrm{CES}-D/EURO-D}_{third} \right)/2\times{time}_{third-second}$$

$$\mathrm{Cum}A\mathrm{DS}=\left[ \left( {\mathrm{CES}-D/EURO-D}_{first}+{\mathrm{CES}-D/EURO-D}_{second} \right)/2\times{time}_{second-first}+ \left( {\mathrm{CES}-D/EURO-D}_{second}+{\mathrm{CES}-D/EURO-D}_{third} \right)/2\times{time}_{third-second} \right]/{time}_{third-first}$$

$$Cumulative burden=\left[ \left( {\mathrm{CES}-D/EURO-D}_{first}+{\mathrm{CES}-D/EURO-D}_{second} \right)/2-cutoff \right]\times{time}_{second-first}+ \left[ \left( {\mathrm{CES}-D/EURO-D}_{second}+{\mathrm{CES}-D/EURO-D}_{third} \right)/2-cutoff \right]\times{time}_{third-second}$$

Where ${\mathrm{CES}-D/EURO-D}_{first}$, ${\mathrm{CES}-D/EURO-D}_{second}$, and ${\mathrm{CES}-D/EURO-D}_{third}$ indicated depression symptoms scores at baseline, the second examinations, and the third examination, ${time}_{second-first}$, ${time}_{third-second}$ and ${time}_{third-first}$indicated the participant-specific time interval between consecutive examinations in years.


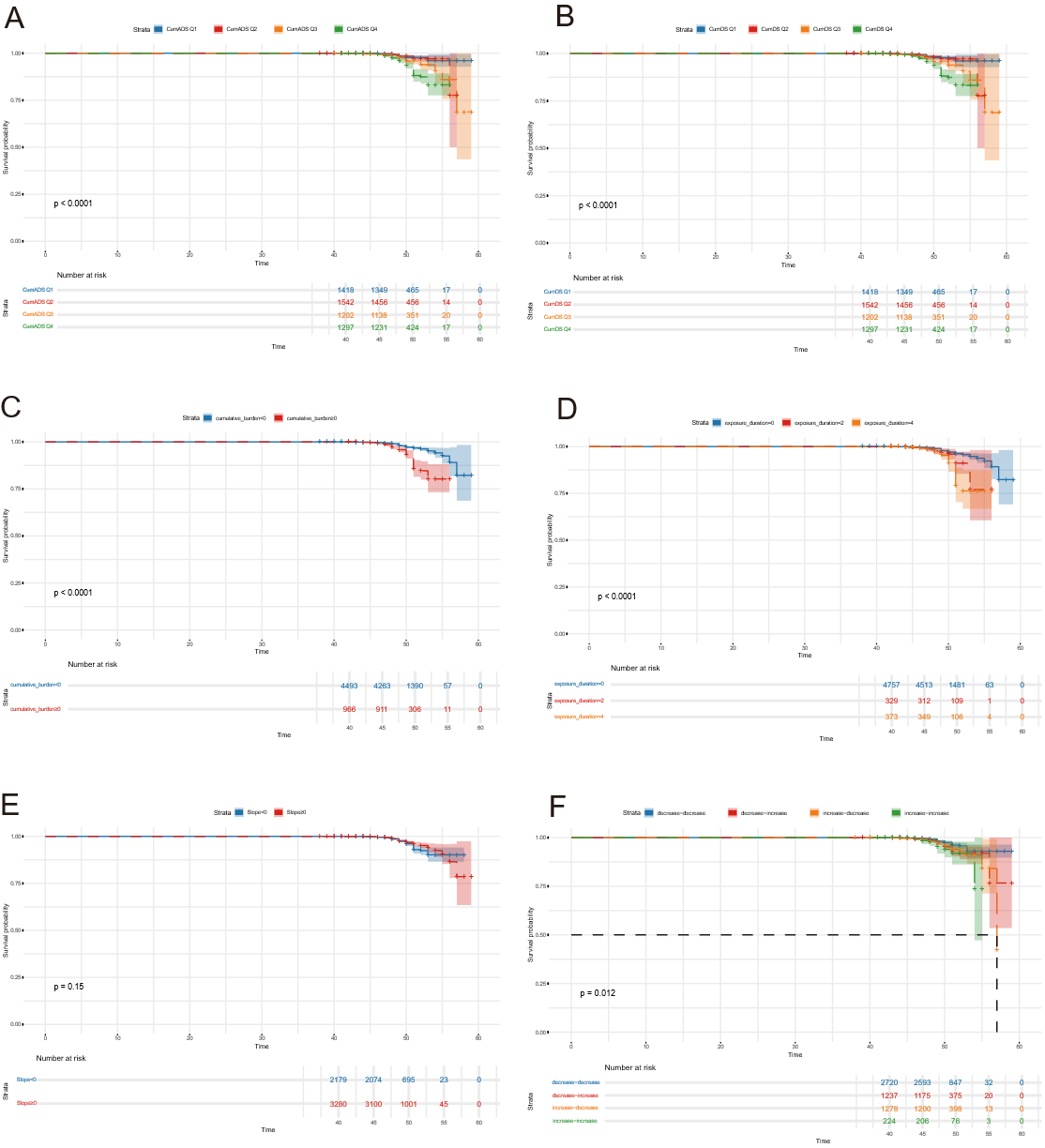


Supplementary Figure 1 Kaplan–Meier curves for CumADS, CumDS, cumulative burden, exposure duration, slope, and time course patterns in ELSA.

CumADS(A), CumDS(B), cumulative burden(C), exposure duration(D), slope(E), and time course patterns(F)


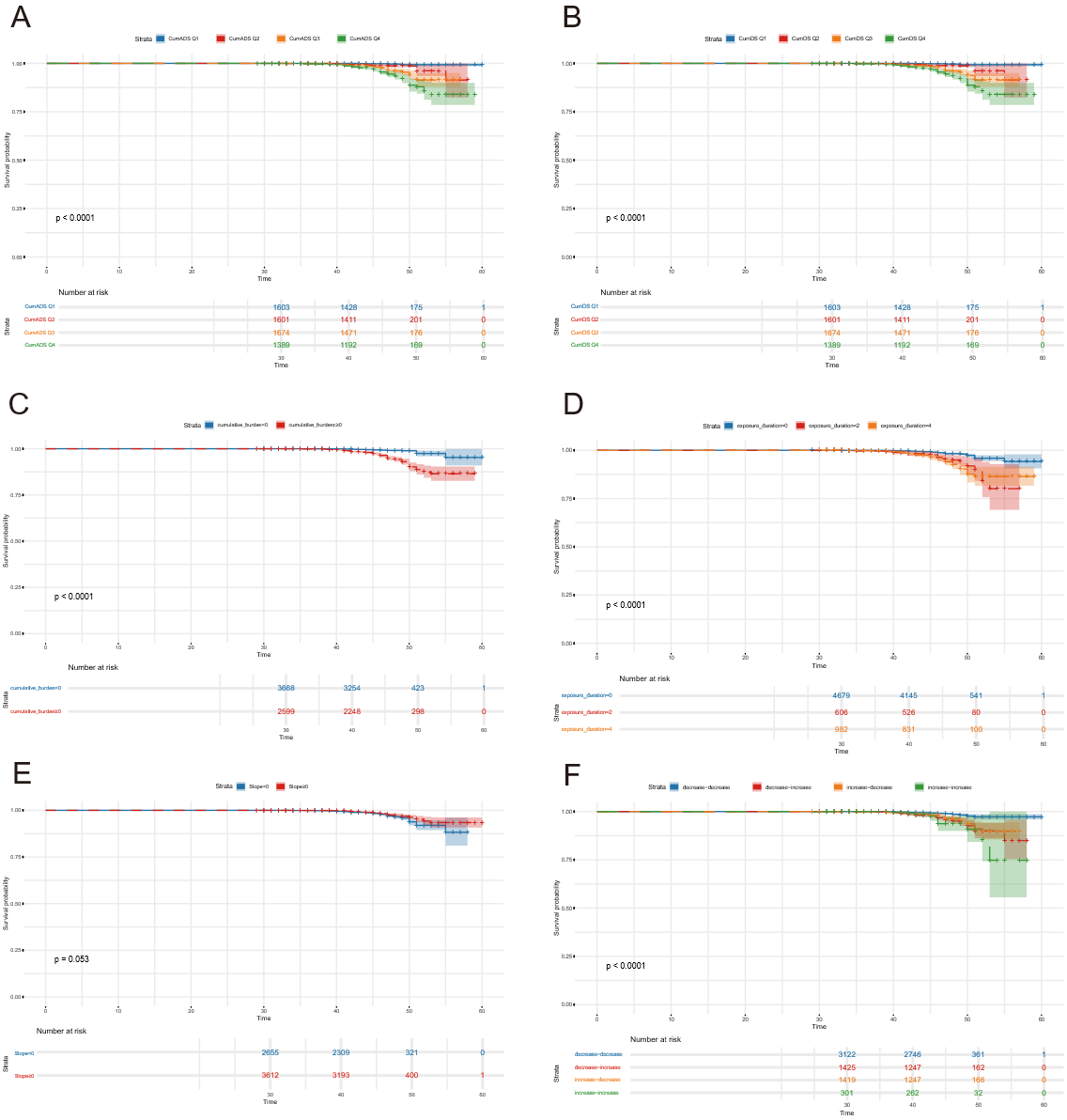


Supplementary Figure 2 Kaplan–Meier curves for CumADS, CumDS, cumulative burden, exposure duration, slope, and time course patterns in HRS.

CumADS(A), CumDS(B), cumulative burden(C), exposure duration(D), slope(E), and time course patterns(F)


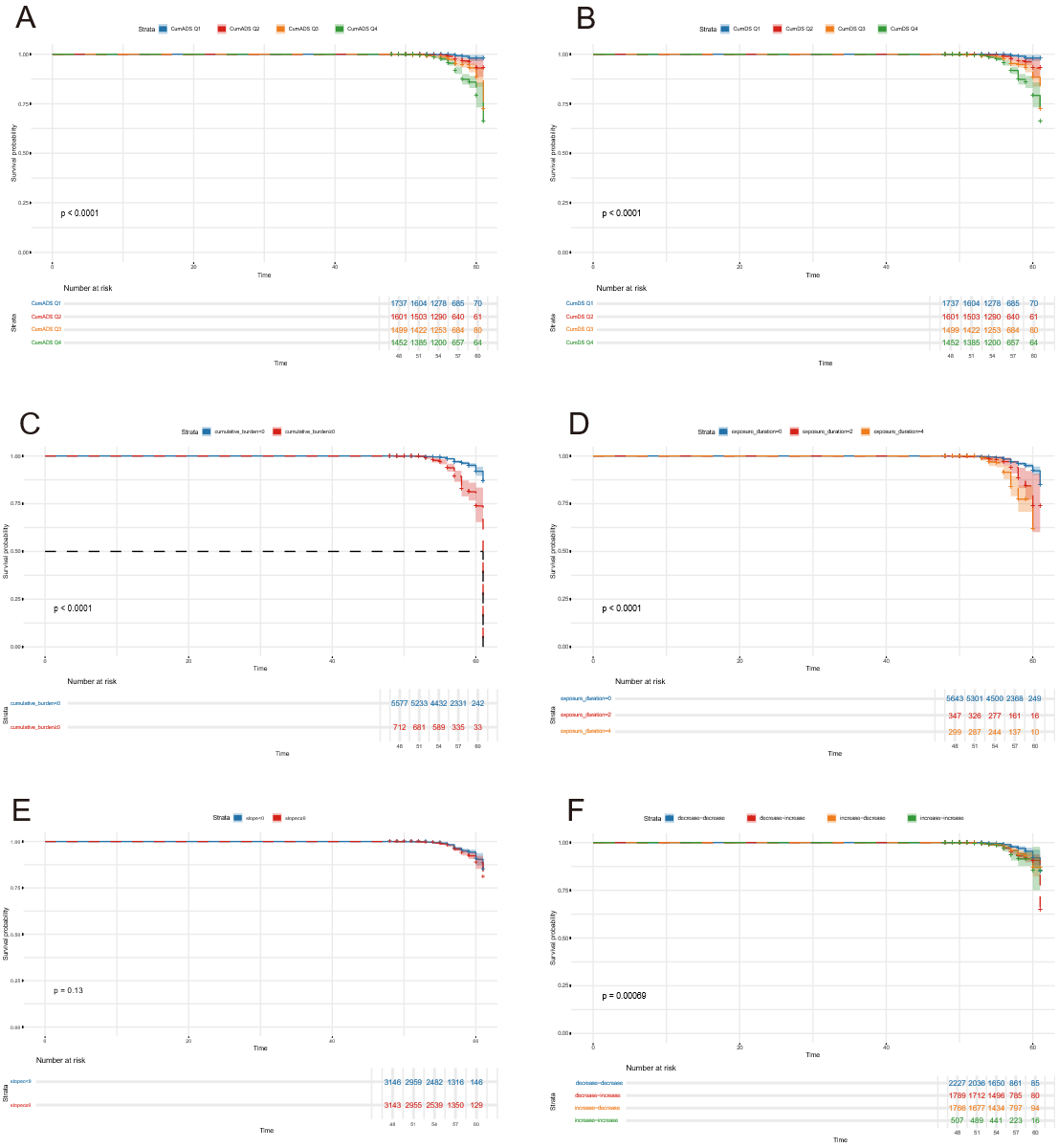


Supplementary Figure 3 Kaplan–Meier curves for CumADS, CumDS, cumulative burden, exposure duration, slope, and time course patterns in SHARE.

CumADS(A), CumDS(B), cumulative burden(C), exposure duration(D), slope(E), and time course patterns(F)


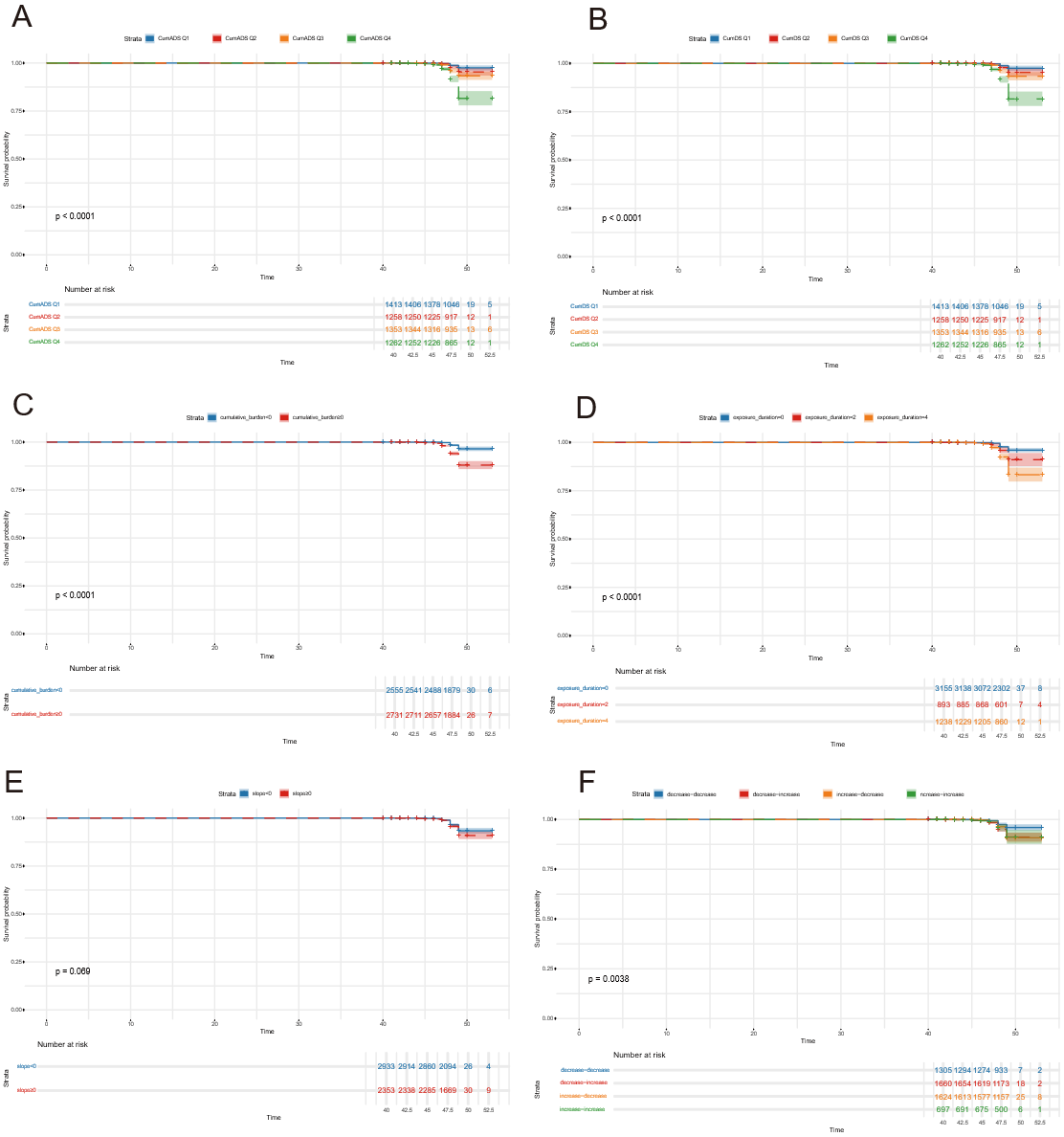


Supplementary Figure 4 Kaplan–Meier curves for CumADS, CumDS, cumulative burden, exposure duration, slope, and time course patterns in CHARLS.

CumADS(A), CumDS(B), cumulative burden(C), exposure duration(D), slope(E), and time course patterns(F)
